# Longitudinal microstructural alterations surrounding subcortical ischemic stroke lesions detected by free‐water imaging

**DOI:** 10.1002/hbm.26722

**Published:** 2024-05-23

**Authors:** Felix L. Nägele, Marvin Petersen, Carola Mayer, Marlene Bönstrup, Robert Schulz, Christian Gerloff, Götz Thomalla, Bastian Cheng

**Affiliations:** ^1^ Department of Neurology University Medical Center Hamburg‐Eppendorf Hamburg Germany; ^2^ Department of Neurology University of Leipzig Medical Center Leipzig Germany

**Keywords:** diffusion MRI, free‐water imaging, penumbra, small vessel disease, subcortical stroke

## Abstract

In this study we explore the spatio‐temporal trajectory and clinical relevance of microstructural white matter changes within and beyond subcortical stroke lesions detected by free‐water imaging. Twenty‐seven patients with subcortical infarct with mean age of 66.73 (SD 11.57) and median initial NIHSS score of 4 (IQR 3–7) received diffusion MRI 3–5 days, 1 month, 3 months, and 12 months after symptom‐onset. Extracellular free‐water and fractional anisotropy of the tissue (FA_T_) were averaged within stroke lesions and the surrounding tissue. Linear models showed increased free‐water and decreased FA_T_ in the white matter of patients with subcortical stroke (lesion [free‐water/FA_T_, mean relative difference in %, ipsilesional vs. contralesional hemisphere at 3–5 days, 1 month, 3 months, and 12 months after symptom‐onset]: +41/−34, +111/−37, +208/−26, +251/−18; perilesional tissue [range in %]: +[5–24]/−[0.2–7], +[2–20]/−[3–16], +[5–43]/−[2–16], +[10–110]/−[2–12]). Microstructural changes were most prominent within the lesion and gradually became less pronounced with increasing distance from the lesion. While free‐water elevations continuously increased over time and peaked after 12 months, FA_T_ decreases were most evident 1 month post‐stroke, gradually returning to baseline values thereafter. Higher perilesional free‐water and higher lesional FA_T_ at baseline were correlated with greater reductions in lesion size (rho = −0.51, *p* = .03) in unadjusted analyses only, while there were no associations with clinical measures. In summary, we find a characteristic spatio‐temporal pattern of extracellular and cellular alterations beyond subcortical stroke lesions, indicating a dynamic parenchymal response to ischemia characterized by vasogenic edema, cellular damage, and white matter atrophy.

## Graphical abstract

This longitudinal study shows a characteristic spatio‐temporal pattern of extracellular and cellular white matter alterations within the first 12 months after subcortical stroke extending well beyond visible lesions. These findings indicate a dynamic parenchymal response to cerebral ischemia characterized by vasogenic edema, cellular damage, and white matter atrophy.

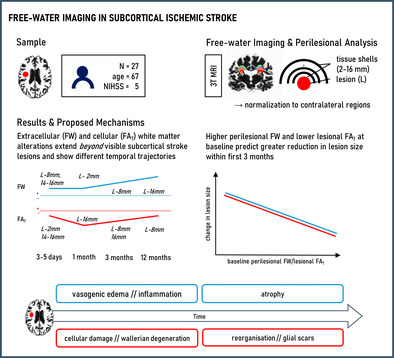

## 1 INTRODUCTION

Ischemic stroke is among the leading causes of disability worldwide (Feigin et al., 2021). Although lesion volume is a significant predictor of long‐term outcomes (Vogt et al., 2012), it is well known that small, yet strategically located subcortical infarcts may just as well lead to debilitating impairments such as paresis (Cheng et al., 2020; Liu et al., 2020) or cognitive decline (Munsch et al., 2016).

While approximately 20%–30% of ischemic strokes are attributable to subcortical infarcts, their pathophysiology remains incompletely understood, not least because of the complex interplay between endothelial dysfunction, blood–brain barrier disruption, neuroinflammation, and subsequent ischemic injury (Regenhardt et al., 2018; Wardlaw et al., 2019). In an effort to elucidate mechanisms of damage and repair which may ultimately be translated into specific treatment and prevention approaches, investigations of local tissue properties have recently gained attention (Regenhardt et al., 2018; van den Brink et al., 2023; Wardlaw et al., 2019). Neuroimaging has proven a powerful tool in furthering our understanding of in vivo brain structural changes associated with ischemic stroke (Guggisberg et al., 2019; Mandeville et al., 2017). In particular, diffusion‐weighted magnetic resonance imaging (MRI) has been used extensively to describe degenerative alterations of white matter tracts following an ischemic event (Moura et al., 2019). Various diffusion tensor imaging (DTI) studies show that white matter degeneration occurs not only at the site of injury, but also in distant areas during the weeks after stroke (Buch et al., 2016; Cheng et al., 2021; Crofts et al., 2011; Radlinska et al., 2010; Thomalla et al., 2004). The latter phenomenon has been interpreted as imaging evidence of Wallerian degeneration and, in the case of the corticospinal tract (CST), for example, has been shown to be correlated with motor function (Thomalla et al., 2004, 2005). Whereas these tract‐specific investigations provide plausible and important neuroanatomical explanations for structural brain changes and clinical outcomes, the role of the local tissue environment within and surrounding the primary ischemic lesion remains poorly understood.

Recently, more advanced diffusion MRI models, such as free‐water imaging, have been proposed to overcome methodological limitations of biological interpretability of conventional DTI metrics like fractional anisotropy (FA) (Dhollander et al., 2021; Pasternak et al., 2009). Free‐water imaging models two compartments: the first compartment reflects the volume fraction of isotropic diffusion of extracellular free‐water at 37°C which may be sensitive to vasogenic edema or enlarged extracellular spaces due to atrophy (Di Biase et al., 2021; Ofori et al., 2019; Pasternak et al., 2009). The second compartment models hindered/restricted diffusion in the proximity of cellular membranes of which FA of the tissue (FA_T_) can be estimated. Therefore, FA_T_ more closely reflects the cellular microstructure of axons and myelin than conventional FA (Pasternak et al., 2009). While some studies (Archer et al., 2017; Duering et al., 2020; Guder et al., 2021; Kern et al., 2022) have established free‐water imaging as a useful tool to investigate stroke‐related pathology, more fine grained *longitudinal* studies probing local cellular and extracellular changes in subcortical lesions and the surrounding tissue are lacking. However, such studies characterizing “penumbral” white matter alterations may provide important pathophysiological information which may ultimately be used to alter tissue fate and thus recovery trajectories in this relevant stroke population.

Utilizing free‐water imaging, we therefore aimed to study cellular (FA_T_) and extracellular (free‐water) changes within and surrounding subcortical stroke lesions from 3 to 5 days up to 12 months after the ischemic event in a well‐defined longitudinal sample of subcortical stroke patients. Based on previous work studying microstructural changes related to small vessel disease and stroke, we hypothesized that we find white matter alterations extending beyond lesions visible on conventional MRI (Kern et al., 2022; Mayer et al., 2022; Radlinska et al., 2010; Thomalla et al., 2005). Moreover, we evaluated associations of lesional and perilesional diffusion imaging markers with clinical measures and longitudinal change in stroke volume.

## 2 MATERIALS AND METHODS

Reporting follows the Strengthening the Reporting of Observational Studies in Epidemiology (STROBE) guideline (Vandenbroucke et al., 2007).

### 2.1 Ethics

The current study was approved by the local ethics committee (Landesärztekammer Hamburg [State of Hamburg Chamber of Medical Practitioners]) and all participants gave written informed consent. The study has been conducted in accordance with the current revision of the Declaration of Helsinki and Good Clinical Practice.

### 2.2 Study population

We have previously reported on data of this longitudinal stroke cohort which was collected within the Collaborative Research Centre 936 between 2012 and 2017 (Cheng et al., 2020, 2021). Patients were recruited via the stroke unit of the University Medical Center Hamburg‐Eppendorf 3–5 days after their first‐time ischemic stroke in case they met the following inclusion criteria: (1) upper limb motor deficit; (2) isolated acute ischemic stroke lesion and absence of obvious white matter lesions or lesions from previous stroke determined by MRI; (3) written informed consent; (4) absence of severe neurological or non‐neurological co‐morbidity. Exclusion criteria were conditions of pre‐existing structural brain damage, for example, previous stroke, intracranial hemorrhage, any other space‐occupying lesion, as determined by fluid attenuated inversion recovery (FLAIR) and T1‐weighted images. Upper limb motor deficits were defined as arm paresis or reduced finger dexterity as reported by the patient and confirmed in clinical examination. The focus on patients with upper limb impairment takes into account the significant effect thereof on activities of daily living and its role as a key target in rehabilitative research (Cordes et al., 2024). MRI and clinical examinations of patients were conducted at four time points: during the acute phase (3–5 days post‐stroke), as well as in the subacute and chronic phases, that is, 30–40 days (~ 1 month), 85–95 days (~3 months), and 340–380 days (~12 months) after stroke. For the current analysis, only patients with isolated subcortical infarcts were included. A map showing the lesion distribution of the study sample is shown in Figure S1.

### 2.3 Clinical testing

Clinical examinations included the National Institute of Health Stroke Scale (NIHSS), the modified Rankin scale (mRS), the Fugl‐Meyer assessment of the upper extremity (UEFM), nine hole peg test (NHP), as well as grip force of the affected hand (in kg), calculated as the mean of three consecutive measurements, relative to the unaffected hand.

### 2.4 Image acquisition

Scanning was performed on a single 3T Siemens Skyra MRI scanner (Siemens, Erlangen, Germany). Imaging sequences included high‐resolution T1‐weighted anatomical, FLAIR, and diffusion weighted images (DWI) with whole‐brain coverage and 2 × 2 × 2 mm resolution, acquired along 64 non‐collinear gradient directions (*b* = 1500 s/mm^2^) and one image with *b* = 0 s/mm^2^. The *b*‐value of 1500 was chosen to enhance the sensitivity to microstructural changes associated with ischemic stroke and to allow for advanced diffusion modelling compared to lower *b*‐values, while still achieving a reasonable signal‐to‐noise ratio (SNR) (Dhollander et al., 2021; Jones et al., 2013; Tournier et al., 2013). Detailed acquisition parameters can be found in Appendix S1.

### 2.5 Image processing

**FIGURE 1:**
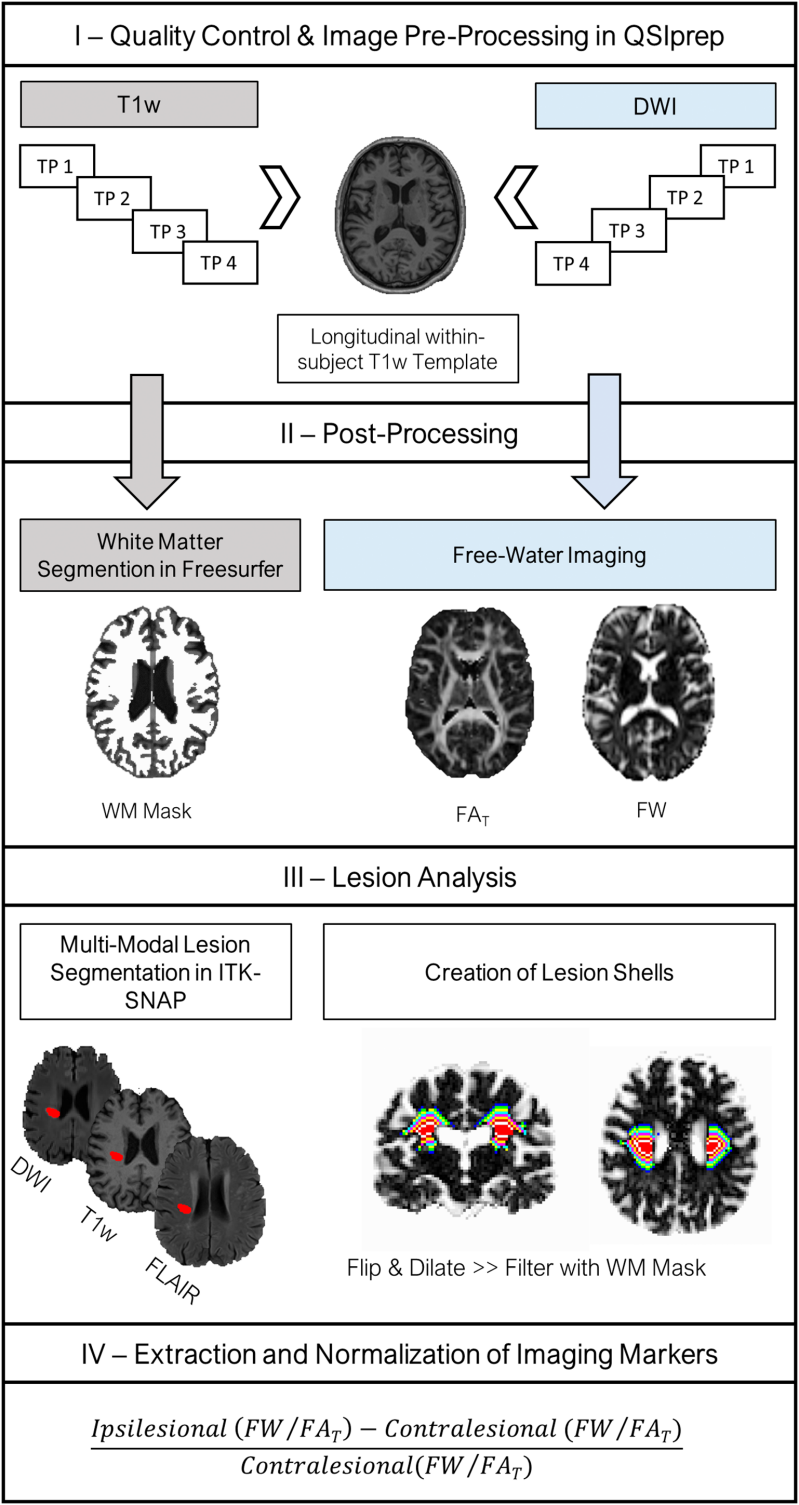
Overview of the imaging pipeline. Schematically illustrates the basic steps of the imaging pipeline used for analysis. DWI, diffusion‐weighted images; FA_T_, fractional anisotropy of the tissue; FLAIR, fluid‐attenuated inversion recovery images; FW, free‐water; T1w, T1‐weighted images; TP1, 3–5 days; TP2, 1 month; TP3, 3 months; TP4, 12 months after stroke; WM, white matter.

An overview of the imaging pipeline can be found in Figure 1. All code is publicly available on GitHub: https://github.com/csi-hamburg/CSIframe. After visual quality control of raw data, preprocessing of anatomical T1w and DWI was conducted in *QSIPrep* 0.14.2 (Cieslak et al., 2021), resulting in within‐subject co‐registered DWI and T1w images for each subject and time point. A detailed description of image preprocessing can be found in Appendix S1.

#### 2.5.1 Free‐water imaging

Free‐water imaging, a two‐tensor diffusion MRI model, was used to model an extracellular compartment of isotropic diffusion, as well as a cellular compartment characterized by hindered/restricted diffusion of water molecules (Pasternak et al., 2009). Thus, by means of a regularized non‐linear fit of preprocessed DWIs, the volume fraction of free‐water, and free‐water corrected diffusion tensors were estimated at the voxel level for each study participant and time point. A customized *MATLAB* script developed by Dr. Pasternak based on the original free‐water imaging model (Pasternak et al., 2009) was used for the bi‐tensor fit (The MathWorks, Inc. *MATLAB*. Version 2019b). Finally, maps of fractional anisotropy of the tissue compartment (FA_T_) were derived based on the diffusion tensors (Pasternak et al., 2009).

#### 2.5.2 Lesion analysis

We performed multi‐model semi‐automatic lesion segmentation in *ITK‐SNAP* (Yushkevich et al., 2006) (http://www.itksnap.org/) based on T1w and FLAIR signal alterations. DWI images were used complementary to guide the identification of stroke lesions. Lesions were segmented in T1w space with a clustering approach using linearly co‐registered and preprocessed images of each modality. Details on quality assurance can be found in Appendix S1.

Further, for later normalization of free‐water imaging markers, lesion masks were flipped to the contralateral hemisphere. This was conducted in standard space (MNI152NLin2009cAsym) to ensure anatomical correspondence. Flipped lesion masks were then back transformed to native T1w space for further processing. Original and flipped lesion masks, as well as *FreeSurfer* white matter and ventricle segmentations (derived through *QSIPrep*) were resampled to the DWI voxel size (2 × 2 × 2 mm). The white matter segmentations, which exclude subcortical gray matter, were eroded in two iterations (2 voxels = 4 mm). The ventricle mask was subtracted from the flipped lesion mask to ensure that it does not contain portions of cerebrospinal fluid. Next, eight tissue shells surrounding the original and flipped lesion masks, separately, were created for each time point by iteratively dilating the masks (one iteration = 1 voxel, i.e., 2 mm) in three‐dimensional space and subtracting the previous iterations from the dilated masks. Moreover, corresponding ipsi‐ and contralateral tissue shells were subtracted from each other to avoid overlap. This resulted in 2 × 8 unique tissue shells of increasing distance to the lesion masks with 2 mm width each. In an effort to reduce partial volume effects and to limit the analysis to the cerebral white matter, tissue shells were filtered with individual *FreeSurfer* white matter segmentations. Finally, we extracted average free‐water and FA_T_ for each lesion and tissue shell and normalized the values to the corresponding contralateral, that is, flipped, lesion/tissue shell for further statistical analysis ([ipsilateral – contralateral]/contralateral).

### 2.6 Statistical analysis

All statistical analyses were conducted in R 4.1.3 (https://www.R-project.org/). Testing was performed two‐tailed and the level of significance was set to *p* < .05. Missing data was not imputed. The analysis code can be found on GitHub: https://github.com/felenae/2023_freewater_subcortical_stroke.

#### 2.6.1 Clinical data

Descriptive statistics of clinical characteristics at baseline and 3 months after stroke are presented as mean and standard deviation (SD), median and inter quartile range (IQR), or absolute number and percentage.

#### 2.6.2 Imaging

To characterize the spatial extent of tissue alterations relative to healthy white matter, one‐sample *t*‐tests were performed with normalized diffusion markers for each region of interest and time point, separately.

Next, we fitted longitudinal linear mixed effects models with *subject* and *location of measurement* as random factors to test for differences in normalized free‐water and FA_T_ across time points, adjusting for *lesion volume*, *age*, *sex*, and *days since stroke*. We did not include the raw *days since stroke* variable, but z‐scored it within each time point separately, before entering it into the model, in order to control for deviations in scan interval within the respective time point without eliminating the effect of interest (time point). Here, we used dummy coding for contrasts where the first time point (3–5 days after stroke) served as the reference, and performed Tukey post‐hoc tests for pairwise comparisons between time points.

Finally, to test for differences in normalized imaging markers between the lesion and tissue shells, we fitted additional cross‐sectional linear mixed effects models for each time point. More details and results can be found in Table S2.

#### 2.6.3 Associations between clinical data, change in lesion volume and diffusion markers

We performed Spearman correlations of baseline imaging markers averaged within the lesion and across all tissue shells, separately, with NIHSS, relative grip force, UEFM, and NHP at 3 months after stroke. Moreover, baseline imaging markers were correlated with baseline lesion volume (as a proxy of visible edema extent); as well as with relative change in lesion size from 3–5 days to 1 month, and from 3–5 days to 3 months after stroke.

In case of statistical significance of explorative correlations, we performed post‐hoc linear regressions adjusting for *age*, *sex*, *baseline lesion volume*, and *days since stroke* to test the robustness of the respective finding.

#### 2.6.4 Sensitivity and complementary analyses

To lend further support to the results of the main analyses, we performed additional sensitivity analyses.

First, in order to investigate whether our longitudinal findings were mainly driven by tissue alteration within the lesion, we fitted linear mixed effects models for the lesion and tissue shells, separately, assessing longitudinal change in normalized diffusion measures with *subject* and *location of measurement* as random factors, and *lesion volume*, *age*, *sex*, and *days since stroke* (*z*‐scored) as covariates.

Second, in the light of our relatively small sample size, we sought to validate our main findings in a larger sample by including additional 12 patients with cortical or both cortical and subcortical infarcts, who were assessed with the same study protocol, amounting to a total validation sample size of 39 patients.

Third, as a complementary analysis to support the interpretability and plausibility of alterations in imaging measures, we performed a tract‐of‐interest analysis, investigating longitudinal change in relative free‐water and FA_T_ values of the ipsilateral CST, as well as linear regression analysis of CST tissue alterations with symptom severity (NIHSS) at 3 months after stroke. Further, we related relative free‐water with relative FA_T_ in the CST in the chronic stage, that is, 1 year after stroke. For methodological details and results please refer to Appendix S1.

Last, using the “pwr” package in R, we provide statistical power estimates for our main contrasts of interest, that is, differences in diffusion measures between ipsilesional and contralesional (healthy) tissue, as well as between time points. The results can be found in Table S14.

## 3 RESULTS

### 3.1 Demographics and clinical characteristics

Descriptive statistics are presented in Table 1. DWI data from the original study was available for 52 patients. In total, 27 patients with subcortical ischemic stroke were included in the present analysis. Note that not all 27 participants completed all study visits (3–5 days: *N* = 26, 1 month: *N* = 21, 3 months: *N* = 19, and 12 months: *N* = 19). The participants were on average 67 (SD 11.6) years old and presented with a median baseline NIHSS of 4 (IQR 3–7). The sample was relatively well balanced regarding sex (41% female) and lesion side (44% left side). Numerically, all clinical outcome parameters improved from 3–5 days to 3 months after stroke, while lesion volumes decreased (see also Figure S2). Furthermore, we provide demographical and clinical characteristics stratified by sex in Table S1. Comparing to male patients (*N* = 16), female patients (*N* = 11) were significantly older (74 vs. 61 years, *p* = .002); and, albeit not statistically significant, had larger stroke volumes (11.3 vs. 6.4), as well as a higher baseline NIHSS (median 5 vs. 4).

**TABLE 1:** Sample characteristics.

Variable^a^	Patients with subcortical ischemic stroke
Age in years	66.73 (11.57), 27
Female sex, count (%), *N*	11 (40.7%), 27
Lesion side, left, count (%), *N*	12 (44.4%), 27
Cardiovascular risk factors, count (%), *N*	
Arterial hypertension	22 (91.7), 24
Hypercholesterolemia	10 (41.7), 24
Diabetes	5 (20.8), 24
Smoking	12 (50.0), 24
Obesity	5 (20.0), 24

	3–5 days after stroke	3 months after stroke
Lesion volume in mL^b^	7.72 (11.60), 26	3.76 (5.53), 19
NIHSS, median (IQR), *N*	4 (3–7), 25	1 (0–3), 19
Relative grip strength of affected hand	0.45 (0.40), 22	0.72 (0.40), 18
UEFM	37.92 (26.62), 24	50.79 (23.10), 19
NHP	2.10 (1.51), 12	1.24 (0.32), 14

*Abbreviations*: NHP, nine‐hole‐peg‐test; NIHSS, National Institutes of Health Stroke Scale; UEFM, Fugl‐Meyer assessment of the upper extremity.

^a^ If not specified otherwise, data are presented as mean (standard deviation [SD]), *N*.

^b^ Of patients with available DWI data at the respective time point. See also Figure S2.

### 3.2 Imaging

#### 3.2.1 Cross‐sectional analyses comparing ipsilateral with contralateral tissue properties

**FIGURE 2:**
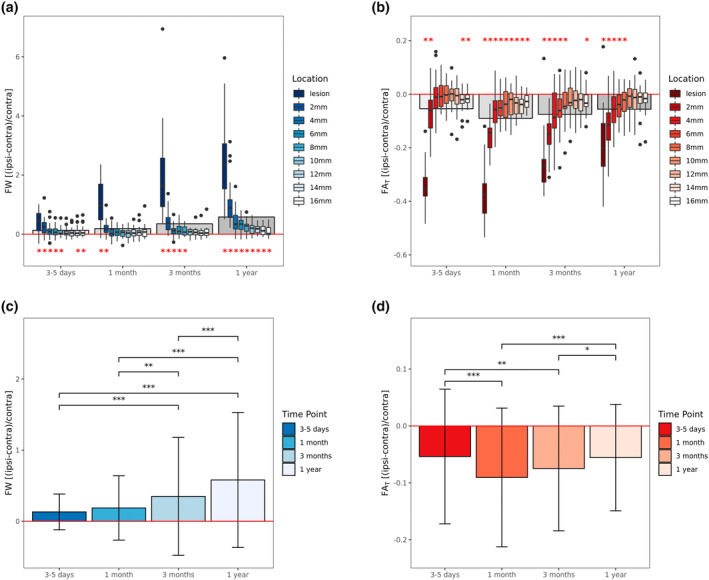
Box plots and bar graphs visualizing relative change in free‐water and FA_T_ by time point and location of measurement. Panels a and b show the relative ipsilateral change in free‐water (a) and FA_T_ (b) compared to contralateral corresponding regions. The box plots are stratified by location of measurement and time point. The lower and upper end of the box corresponds to the 1st and 3rd quartiles, respectively. The line within the box represents the median. The whiskers extend to 1.5 times the interquartile range below and above the 1st and 3rd quartiles, respectively. The red horizontal line indicates the level of zero change, the gray rectangles in the background represent the mean across all regions of interest for each time point. Red asterisks indicate a significant difference from zero change (*p* < .05) as determined by one‐sample *t*‐tests for each region of interest, separately. Panels c and d show the relative ipsilateral change in free‐water (c) and FA_T_ (d) averaged across regions of interest for each time point. The whiskers extend from one standard deviation above to one below the mean. Asterisks indicate the level of significance (***p < .001, **p < .01, *p < .05) derived from longitudinal linear mixed‐effects models and post‐hoc Tukey's tests.

Results of the cross‐sectional analyses investigating differences between ipsilateral and contralateral tissue properties for each time point can be found in Table 2. Boxplots of imaging measures at the four different time points stratified by location of measurement are visualized in Figure 2a,b.

**TABLE 2:** Results of one‐sample *t*‐tests for each time point, investigating whether ipsilesional diffusion parameters are different compared to corresponding contralateral regions.

Location	Relative free‐water			Relative FA_T_		
	Mean % [95% CI]	*d* ^a^	*p*	Mean % [95% CI]	*d* ^a^	*p*
3–5 days (*N* = 26)						
Lesion	40.6 [24.4, 56.8]	1.43	<.001***	−34.3 [−37.4, −31.2]	−6.28	<.001***
2 mm	24.0 [12.2, 35.8]	1.16	<.001***	−6.9 [−10.0, −3.7]	−1.25	<.001***
4 mm	10.1 [0.2, 19.9]	0.58	.05*	−0.9 [−3.8, 2.1]	−0.17	.54
6 mm	8.1 [1.2, 15.0]	0.67	.02*	−0.4 [−2.6, 1.8]	−0.11	.69
8 mm	7.6 [1.8, 13.4]	0.75	.01*	−0.2 [−2.0, 1.6]	−0.07	.81
10 mm	5.3 [−0.9, 11.5]	0.49	.09	−0.6 [−2.4, 1.3]	−0.18	.53
12 mm	5.9 [−0.9, 12.7]	0.49	.09	−1.3 [−3.5, 0.8]	−0.35	.21
14 mm	7.4 [0.04, 14.8]	0.57	.05*	−2.1 [−3.6, −0.6]	−0.78	.010**
16 mm	9.2 [2.0, 16.5]	0.73	.01*	−1.7 [−2.9, −0.5]	−0.79	.009**
1 month (*N* = 21)						
Lesion	111.2 [77.6, 144.9]	2.13	<.001***	−37.3 [−42.0, −32.6]	−5.11	<.001***
2 mm	20.1 [7.7, 32.6]	1.04	.003**	−15.6 [−18.2, −12.9]	−3.73	<.001***
4 mm	3.5 [−7.2, 14.3]	0.21	.50	−6.6 [−9.6, −3.5]	−1.38	<.001***
6 mm	5.7 [−1.9, 13.3]	0.48	.13	−4.5 [−7.0, −1.9]	−1.12	.002**
8 mm	3.7 [−3.7, 11.0]	0.32	.31	−3.7 [−6.3, −1.0]	−0.89	.009**
10 mm	2.4 [−5.2, 9.9]	0.20	.52	−3.6 [−5.9, −1.4]	−1.05	.003**
12 mm	5.5 [−2.6, 13.5]	0.43	.17	−3.7 [−5.8, −1.6]	−1.12	.002**
14 mm	7.2 [−2.0, 16.5]	0.50	.12	−3.9 [−5.5, −2.2]	−1.50	<.001***
16 mm	9.1 [−1.8, 20.0]	0.54	.10	−2.7 [−4.1, −1.4]	−1.28	<.001***
3 months (*N* = 19)						
Lesion	207.7 [132.3, 283.0]	1.88	<.001***	−25.9 [−31.6, −20.3]	−3.13	<.001***
2 mm	43.4 [25.0, 61.9]	1.61	<.001***	−15.5 [−19.5, −11.6]	−2.69	<.001***
4 mm	13.0 [2.4, 23.6]	0.83	.02*	−8.2 [−13.0, −3.3]	−1.15	.002**
6 mm	14.1 [4.0, 24.2]	0.95	.009**	−5.6 [−9.7, −1.4]	−0.91	.01*
8 mm	10.7 [3.2, 18.2]	0.98	.008**	−3.5 [−6.9, −0.1]	−0.69	.0470*
10 mm	5.0 [−1.0, 11.1]	0.57	.09	−2.2 [−5.4, 1.0]	−0.46	.17
12 mm	7.4 [−0.6, 15.4]	0.63	.07	−1.8 [−4.7, 1.1]	−0.43	.20
14 mm	5.2 [−3.2, 13.5]	0.42	.21	−2.2 [−4.9, 0.5]	−0.55	.11
16 mm	7.9 [−2.5, 18.4]	0.52	.13	−2.4 [−4.9, < 0]	−0.68	.0496*
12 months (*N* = 19)						
Lesion	251.3 [180.3, 322.3]	2.41	<.001***	−18.3 [−25.0, −11.6]	−1.87	<.001***
2 mm	109.4 [69.7, 149.0]	1.88	<.001***	−11.8 [−16.4, −7.3]	−1.77	<.001***
4 mm	44.3 [22.9, 65.8]	1.41	<.001***	−5.3 [−9.3, −1.4]	−0.92	.01*
6 mm	33.7 [20.0, 47.3]	1.69	<.001***	−4.2 [−7.0, −1.4]	−1.01	.006**
8 mm	24.9 [14.5, 35.4]	1.63	<.001***	−3.1 [−5.9, −0.3]	−0.74	.03*
10 mm	18.3 [8.5, 28.2]	1.27	.001**	−0.021 [−4.8, 0.5]	−0.55	.11
12 mm	17.5 [9.8, 25.3]	1.54	<.001***	−1.3 [−4.4, 1.8]	−0.29	.38
14 mm	13.5 [5.3, 21.7]	1.13	0.003**	−1.9 [−4.7, 0.8]	−0.48	.16
16 mm	9.6 [1.7, 17.5]	0.83	.02*	−1.9 [−4.4, 0.5]	−0.54	.11

Abbreviations: CI, confidence interval; FA_T_, fractional anisotropy of the tissue.

* *p* < .05;

** *p* < .01;

*** *p* < .001.

^a^ Cohen's *d*.

As compared to healthy tissue, 3–5 days after stroke, free‐water was significantly increased in the lesion, 2–8 mm, as well as 14–16 mm tissue shells. The most prominent increase compared to the contralesional hemisphere occurred in the lesion with +40.6% (*p* < .001), gradually decreasing to +5.3% (*p* = .09) at 10 mm distance, increasing again to +9.2% (*p* = .01) at 16 mm distance. On the other hand, relative FA_T_ was decreased ipsilesionally comparing to contralesional healthy tissue. Significant reductions were found in the lesion (−34.3%, *p* < .001), at 2 mm (−6.9%, *p* < .001), 14 mm (−2.1%, *p* = .01), and 16 mm distance (−1.7%, *p* = .009).

One month after stroke, free‐water alterations were less widespread with significant increases being only found in the lesion (+111.2%, *p* < .001) and 2 mm tissue shell (+20.1%, *p* = .003). In contrast, FA_T_, was significantly decreased in all regions of interest with a continuous gradient from −37.3% (*p* < .001) in the lesion to −2.7% (*p* < .001) at 16 mm distance.

At the third imaging assessment 3 months after stroke, significant free‐water increases occurred in the lesion (+207.7%, *p* < .001) and at 2–8 mm distance ranging from +43.4% to +10.7% (*p* < .05), continuously decreasing with further distance. At the same time, FA_T_ decreases were less widespread comparing to 1 month after stroke. FA_T_ was significantly reduced by −25.9% in the lesion (*p* < .001), −15.5% at 2 mm (*p* < .001), −8.2% at 4 mm (*p* = .002), −5.6% at 6 mm (*p* = .01), −3.5% at 8 mm (*p* = .047) and by −2.4% at 16 mm distance (*p* = .0496).

Finally, at the last imaging examination 12 months after stroke, significant free‐water alterations encompassed all regions of interest ranging from +251.3% increase in the lesion (*p* < .001) to +9.6% increase at 16 mm distance (*p* = .02). The spatial extent of FA_T_ reductions was comparable to the previous time point, that is, FA_T_ was decreased by −18.3% in the lesion (*p* < .001), −11.8% at 2 mm (*p* < .001), −5.3% at 4 mm (*p* = .01), −4.2% at 6 mm (*p* = .006) and by −3.1% at 8 mm distance (*p* = .03).

#### 3.2.2 Longitudinal analyses comparing ipsilateral relative free‐water and FA_T_ between time points

Detailed results of the longitudinal linear mixed effects models testing for differences in relative free‐water and FA_T_ between time points are shown in Table 3 and visualized in Figure 2c,d.

**TABLE 3:** Results of the longitudinal linear mixed effects models investigating differences in free‐water and FA_T_ between different time points.

	Free‐water		FA_T_	
	0.498/0.075		0.681/0.020	
*R* ^2^ conditional/marginal^a^	Estimate (SE)	*p*	Estimate (SE)	*p*
Intercept^b^	0.693 (0.286)	.02*	−0.054 (0.061)	.39
Lesion volume^b^	−0.003 (0.003)	.33	<−0.001 (<0.001)	.59
Age^b^	−0.007 (0.003)	.04*	<−0.001 (0.001)	.82
Sex (male)^b^	−0.149 (0.070)	.05*	0.017 (0.015)	.29
Days since stroke, *z*‐scored within time point^b^	−0.012 (0.019)	.53	0.003 (0.003)	.29
Time point^b^		<.001***		<.001***
1 month to 3–5 days^c^	0.049 (0.050)	.76	−0.036 (0.007)	<.001***
3 months to 3–5 days^c^	0.225 (0.053)	<.001***	−0.026 (0.007)	.002**
1 year to 3–5 days^c^	0.449 (0.053)	<.001***	−0.008 (0.007)	.72
3 months to 1 month^c^	0.176 (0.053)	.005**	0.010 (0.007)	.52
1 year to 1 month^c^	0.400 (0.053)	<.001***	0.028 (0.007)	<.001***
1 year to 3 months^c^	0.225 (0.053)	<.001***	0.019 (0.007)	.05*

Abbreviations: FA_T_, fractional anisotropy of the tissue; SE, standard error.

* *p* < .05;

** *p* < .01;

*** *p* < .001.

^a^ Conditional: proportion of variance explained by both the fixed and random effects in the model; marginal: proportion of variance explained by the fixed effects in the model.

^b^ Linear mixed‐effects models with diffusion parameter as dependent variable, time point, lesion volume, age, sex and days since stroke (*z*‐scored within time point) as fixed effects, and subject, as well as location of measurement as random intercept.

^c^ Post‐hoc Tukey's tests comparing different time points with one another.

*Time point* was a significant predictor of both relative free‐water (*Χ* ^2^ = 91.0(3), *p* < .001) and FA_T_ (*Χ* ^2^ = 32.4(3), *p* < .001). Relative free‐water increased continuously over time and was most prominent 1 year after stroke. Post‐hoc Tukey tests revealed that all time points were significantly different from each other (*p* < .001), with the exception of 3–5 days compared to 1 month after stroke. The temporal dynamics of FA_T_ alterations were different compared to those of free‐water. More specifically, relative FA_T_ decreases were most pronounced 1 month after stroke, followed by a steady increase, resulting in similarly reduced FA_T_ levels 12 months compared to 3–5 days after stroke. In terms of covariates, higher age and male sex were associated with lower relative free‐water (*β* = −0.007, *p* = .04, *β* = −0.149, *p* < .05, respectively). There was no significant effect of *lesion volume* or *days since stroke (z‐scored)* on either of the two imaging markers in the longitudinal model.

#### 3.2.3 Associations between clinical data, change in lesion volume and diffusion markers

There were no significant associations between diffusion parameters (FW and FA_T_) 3–5 days after stroke and clinical outcomes 3 months later (Table S3).

**FIGURE 3:**
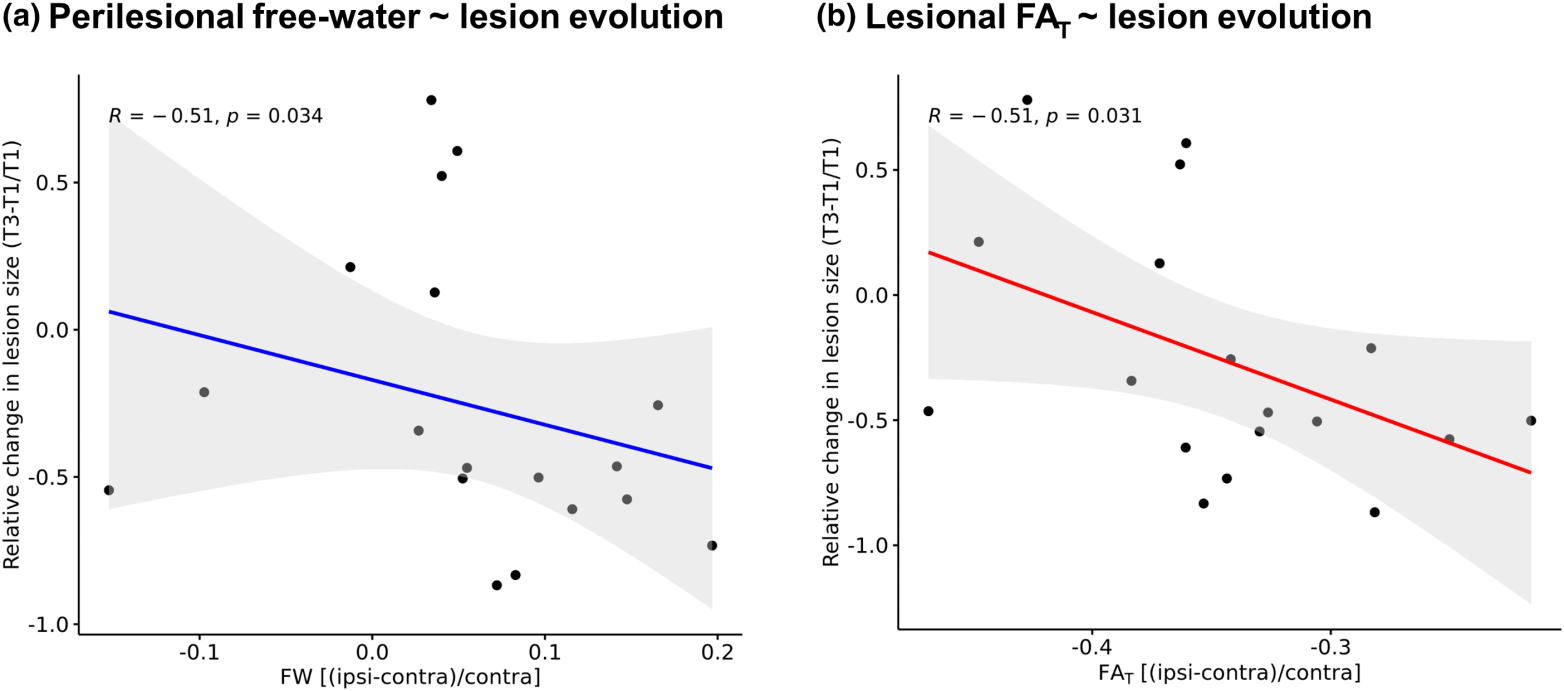
Correlations of baseline perilesional free‐water and lesional FA_T_ with lesion evolution. FA_T_, fractional anisotropy of the tissue; FW, free‐water; T1, 3–5 days (baseline); T3, 3 months after stroke.

However, while 13 out of 18 (72%) lesions became smaller over time, we did observe a significant inverse correlation between perilesional free‐water and change in lesion size from 3–5 days to 3 months after stroke, indicating that greater baseline free‐water increases surrounding the lesion were associated with a greater reduction in lesion size (rho = −0.51, *p* = .03). In addition, higher lesional FA_T_ was significantly associated with greater reductions in lesion size after 3 months (rho = −0.51, *p* = .03) (Figure 3). Associations of relative perilesional/lesional diffusion parameters with change in lesion size did not survive adjustment for covariates in post‐hoc linear regression models (*p*_lesional FAT_ = .12; *p*_perilesional free‐water_ = .74). However, here, male sex was associated with greater reductions in lesion size (*p* = .01).

Baseline relative diffusion parameters did not correlate significantly with baseline lesion volume or change in lesion volume from 3–5 days to 1 month after stroke.

#### 3.2.4 Sensitivity and complementary analyses

Detailed results of the sensitivity and complementary analysis can be found in Appendix S1. Briefly, both sensitivity and validation analyses substantiated consistent results compared to our main analyses, providing further confidence in the robustness of our findings (Figures S3–S6, as well as Tables S4–S13). Additionally, the complimentary analysis showed significant microstructural alterations of the CST with a temporal trajectory analogous to the main analysis (Figure S5, Tables S11 and S12), along with an association of FA_T_ in the CST with NIHSS 3 months after stroke (Figure S6). Lastly, in the chronic stage 1 year after stroke, relative free‐water in the CST but not in the lesion/perilesional tissue was correlated with relative FA_T_ in the CST (Table S13).

## 4 DISCUSSION

In a longitudinal free‐water imaging study over 12 months after subcortical ischemic stroke, we identified dynamic changes of cellular and extracellular white matter alterations most obviously within, but also beyond the primary stroke lesion. Free‐water elevations—already evident at the initial assessment 3–5 days after stroke—continuously increased throughout the study period and were most prominent 12 months after stroke, affecting the entire surrounding tissue under investigation. Cellular changes followed a different trajectory: FA_T_ decreases were most pronounced 1 month after stroke when they extended to all surrounding tissue shells, that is, up to 16 mm distant to the lesion. Following this minimum, FA_T_ steadily increased again, reaching levels comparable to 3–5 days after stroke with remaining reductions in the lesion and up to 8 mm distance. We did not observe significant correlations between baseline diffusion parameters and clinical outcomes 3 months after stroke. However, higher perilesional free‐water and higher lesional FA_T_ were significantly associated with greater reduction in lesion size within the first 3 months after stroke in unadjusted analyses. Sensitivity analyses conducted separately for the lesion and perilesional tissue, as well as validation analyses in a larger sample (*N* = 39), additionally including patients with cortical infarcts, provided consistent results.

### 4.1 Free‐water alterations

Our finding of increased extracellular free‐water beyond visible lesions on conventional structural images, such as T1w and FLAIR images, is in line with previous reports on ischemic stroke patients in the acute to subacute (Kern et al., 2022), as well as chronic phase (Archer et al., 2017; Guder et al., 2021). Archer et al. (2017) assessed patients at least 6 months after stroke in a cross‐sectional, region of interest design and found increased relative free‐water in the ipsilesional primary and premotor CST. Similar to our study, Kern et al. (2022) employed a longitudinal approach comparing global free‐water, averaged across the entire white matter, between four different time points (acute, 24 h, 5 days, and 1 month). Interestingly, they reported an initial free‐water increase outside of the lesion within 24 h, but did not show differences between 5 days and 1 month which is in line with the results of our longitudinal linear mixed effects model. However, our study extends this previous work in that we followed a more fine‐grained approach and continued to map the trajectory up to 1 year after stroke. We found that free‐water elevations relative to corresponding contralateral regions were present at each time point, although to a varying spatial extent with a progressive increase occurring subsequent to the 1‐month assessment.

Various pathological mechanisms may be considered in explaining the observed lesional and perilesional extracellular free‐water elevations. In the early stages of ischemic stroke, vasogenic edema develops in and around the infarct core, eventually becoming visible on T2‐weighted FLAIR images within the first few hours after symptom onset (Thomalla et al., 2011). The free‐water metric measures the amount of freely diffusing water molecules in the extracellular space and may thus be a sensitive marker of vasogenic edema due to upregulated immune responses and consecutive blood–brain‐barrier leakage (Altendahl et al., 2020; Di Biase et al., 2021; Farrher et al., 2021; Pasternak et al., 2009). It is therefore conceivable that the early increases in extracellular diffusivity, which we observe in and beyond the lesion 3–5 days after stroke, are attributable to vasogenic edema and inflammatory processes (Candelario‐Jalil et al., 2022; Chamorro et al., 2012; Kern et al., 2022). These free‐water increases seem to diminish in the surrounding tissue within the first month, suggesting the regredience of edema in this time period. On the other hand, consistent with previous imaging and preclinical histopathological studies (Archer et al., 2017; Cheng et al., 2021; Duering et al., 2020; Farrher et al., 2021), we observed free‐water increases 3 and 12 months after stroke that are most likely explained by secondary mechanisms of neurodegeneration at the microscopic level such as fluid‐filled cavitation and axonal loss (i.e., atrophy) leading to larger extracellular spaces. Further support for this notion comes from our complementary analysis relating increased free‐water to decreased FA_T_ in the CST 1 year after stroke. Intriguingly, free‐water increases were also observed in the normal appearing white matter surrounding the stroke lesion. While our findings fit well with previous studies, more work is needed to strengthen our understanding of the observed spatio‐temporal trajectory of free‐water alterations.

### 4.2 FA_T_ alterations

Compared to free‐water, we observed a different temporal trajectory for FA_T_ suggestive of diverging pathophysiological processes. Similar to free‐water, FA_T_ was altered extending to regions well beyond the visible lesion. The detected reductions in white matter FA_T_ are in line with previous free‐water imaging studies assessing the cellular microstructure of the CST (Archer et al., 2017; Guder et al., 2021). In a previous study, no longitudinal changes in whole‐brain FA_T_ were observed, leading the authors to conclude that FA/FA_T_ might rather capture underlying brain health than stroke‐associated changes (Kern et al., 2022). In contrast, in our sample, relative FA_T_ further decreased from 3–5 days to 1 month after stroke, before returning close to baseline levels after 1 year. These changes may be confined to tissue alterations surrounding the lesion and thus escape a global approach using a whole brain analysis.

Our finding of decreased FA_T_ despite co‐occurring increases in free‐water suggests that reductions in diffusion anisotropy in the proximity of cellular membranes are likely related to damage of myelin sheaths and/or axonal membranes in the cerebral white matter, as previously reported (Marin & Carmichael, 2019). Further, these tissue alterations occurred not only in the lesion, but extended to regions of 16 mm distance, in line with the concept of Wallerian degeneration (Chen et al., 2017; Conforti et al., 2014; Thomalla et al., 2004). This concept is additionally supported by our complimentary tract‐of‐interest analysis which showed FA_T_ reductions of the ipsilesional CST.

Interestingly, FA_T_ reductions compared to the unaffected hemisphere were most prominent 1 month after stroke, followed by a relative “recovery” during the following months. Different potential mechanisms may explain this somewhat surprising trajectory. Initial structural damage caused by ischemia and the following Wallerian degeneration leads to disintegration of axons and surrounding myelin (Buss et al., 2004) indicated by reductions in FA_T_. Thereafter, clearance of cellular debris and the accompanying atrophy of white matter tracts may lead to larger extracellular spaces (as evidenced by free‐water elevations), reduced populations of crossing fibers and a denser configuration of the remaining axons, potentially alleviating FA_T_ reductions (Buss et al., 2004; Cheng et al., 2021). Corroborating this hypothesis, a recent report by our group showed a trend of increasing fiber density—a fixel‐based imaging metric (Dhollander et al., 2021)—in the ipsilateral CST of an overlapping sample (including also non‐subcortical stroke patients) between 1 month and 1 year post‐stroke (Cheng et al., 2021). Lastly, the formation of dense glial scars (Buss et al., 2004; Shen et al., 2021; Zhang et al., 2018) observed several months after cerebral infarction may contribute to higher diffusion anisotropy (Budde et al., 2011) compared to earlier time points. In summary, we observed in vivo evidence of dynamic changes in the lesional and perilesional cellular tissue compartment which are in agreement with established concepts in stroke research. Nevertheless, more research employing similar and different imaging methodologies are needed to further our understanding of the suggested mechanisms.

### 4.3 Consideration of confounding effects

The results of our analyses remained stable, when controlling for potentially confounding effects such as age, sex, and stroke volume. In agreement with a previous report (Rossi et al., 2010), we observed in our cross‐sectional analyses that larger lesion volumes were associated with lower FA_T_ values in the chronic stage, that is, 3 and 12 months after stroke. A finding which is biologically plausible, indicating that greater remaining macroscopic tissue damage is also associated with worse microstructural outcomes.

Interestingly, higher age and male sex was associated with lower relative free‐water in our longitudinal model. While another study has also found that female stroke patients exhibit higher free‐water values (Kern et al., 2022), the association of older age with lower increase in free‐water has not been reported. In contrast, Kern and colleagues reported that free‐water was higher in older patients. Notably, in their model they used absolute free‐water values averaged across the entire white matter (skeleton), whereas here free‐water was expressed as the relative change in relation to the unaffected hemisphere. When correlating age with average free‐water across a white matter skeleton derived by Tract‐Based Spatial‐Statistics (Smith et al., 2006) in our sample (data not presented), we also observe a positive association (rho = 0.29, *p* = .15). In addition, when interpreting age and sex effects in our study, one has to keep in mind that female patients were significantly older than male patients, and that effects became non‐significant in the larger validation sample. Taken together, further research is necessary to elucidate age and sex effects which may be linked to varying parenchymal responses to ischemia.

### 4.4 Associations of imaging measures with clinical outcomes

In our sample of subcortical stroke patients with upper limb motor deficit, we did not observe significant associations of baseline free‐water imaging metrics in the lesion or surrounding tissue with clinical outcomes after 3 months. We believe that the discrepancy to earlier studies (Archer et al., 2017; Guder et al., 2021; Kern et al., 2022) might be due to methodological differences. While our distance‐dependent region of interest approach provided specificity in terms of spatial relation to the lesion, white matter regions not primarily involved in motor networks were included in our analysis, plausibly obscuring structure~(motor) function relationships previously shown in tract‐specific study designs. In line with this explanation, relative FA_T_ measured in the CST was significantly negatively associated with NIHSS 3 months after stroke. Finally, the sample size, especially in the longitudinal analysis, was modest which likely contributed to a lack of power.

On the other hand, similar to a recent study by Duering et al. (2020), we did observe preliminary evidence that free‐water and FA_T_ were associated with the evolution of lesions, although not significantly in adjusted analyses. Higher baseline perilesional free‐water and lesional FA_T_ were associated with greater reduction in lesion size from 3–5 days to 3 months after stroke, while there were no significant associations with baseline lesion volume or change in lesion volume within the first month. In contrast to our study, Duering et al. (2020) did not investigate perilesional changes, but found a trend of lower lesional free‐water in areas that appeared normal on conventional MRI at follow‐up. More research is necessary to establish the clinical significance of altered lesional and perilesional tissue properties detected by diffusion MRI. In the future, quantitative MRI measures indicating stroke‐related tissue changes, such as free‐water, could then potentially be used in proof‐of‐concept treatment trials, comparable to the TREAT‐SVDs trial in small vessel disease (Kopczak et al., 2023).

### 4.5 Strengths and limitations

Strengths of our study include (1) a well‐defined subcortical stroke population, (2) a state‐of‐the‐art, well‐documented and reproducible image processing pipeline, (3) an innovative, fine grained approach to study in vivo white matter tissue properties surrounding stroke lesions, and (4) the longitudinal study design, allowing us to study the longer term dynamics of perilesional microstructural changes.

Nevertheless, several limitations need to be mentioned. While focusing on subcortical stroke may have improved the sensitivity to detect common changes among patients, the generalizability to larger, territorial stroke subtypes remains to be established. Of note, our validation analysis, including patients with cortical stroke, indicates that our findings may be translated to other subtypes of stroke in the future.

Further, we acknowledge that the sample size was small. However, due to the large effect sizes, we were still able to show significant tissue alterations well beyond the stroke lesion with a characteristic temporal trajectory. Our sensitivity and validation analyses lend additional support for the robustness of our results. Taken together, we emphasize the need for larger replication studies, which may show spatially even more extensive, albeit subtle, tissue alterations, and could probe the clinical relevance of perilesional microstructural changes with greater statistical power.

Moreover, we would like to consider the potential impact of the dMRI acquisition on our results. The current study employed single‐shell dMRI with a *b*‐value of 1500 s/mm^2^ and 64 gradient directions. While this relatively high *b*‐value provides several advantages compared to a standard *b*‐value of 1000 s/mm^2^, such as higher angular resolution and smaller measurement error for anisotropy, it comes at cost of a lower SNR especially for the extracellular compartment (Jones et al., 2013; Metzler‐Baddeley et al., 2012; Tournier et al., 2013). Therefore, we used a large number of unique gradient directions and stringent post‐processing techniques (Cieslak et al., 2021) to mitigate potential SNR loss. Future studies may benefit from multi‐shell diffusion MRI data with both lower and higher *b*‐values, allowing an improved fit of the bi‐tensor model and greater sensitivity (Bergmann et al., 2020; Pasternak et al., 2012).

Lastly, our tissue shell approach does not provide tract‐specific information, and may therefore be limited its ability to elucidate brain–behavior relationships.

## 5 CONCLUSION

Utilizing free‐water imaging and a perilesional region of interest approach, our longitudinal study provides novel evidence of dynamic differential extracellular and cellular tissue alterations within *and* beyond subcortical stroke lesions visible on conventional MRI. Mapping the trajectories of these changes, we find that extracellular free‐water elevations, potentially indicative of neuroinflammation and vasogenic edema in the acute phase, decline within the first month, before continuously increasing again in the chronic phase, suggestive of secondary white matter damage. In contrast, FA_T_ reductions peaked in the sub‐acute phase (i.e., after 1 month), indicating that most axonal and myelin damage has occurred by then, whereas later stages may be characterized by microstructural reorganization. External validation of our findings in larger samples including different types of ischemic stroke are needed.

## AUTHOR CONTRIBUTIONS

We describe contributions to the paper using the CRediT contributor role taxonomy. Conceptualization: **F. L. N.**, **G. T.**, **B. C.** Data Curation: **F. L. N.**, **M. P.**, **C. M.**; Formal analysis: **F. L. N.**; Investigation: **F. L. N.**, **M. P.**, **C. M.**, **M. B.**, **R. S.**, **C. G.**, **G. T.**, **B. C.**; Methodology: **F. L. N.**, **C. M.**; Software: **F. L. N.**, **M. P.**, **C. M.**; Supervision: **G. T.**, **B. C.**; Funding Acquisition: **C. G.**, **G. T.**, **B. C.**; Visualization: **F. L. N.**; Writing—original draft: **F. L. N.**; Writing—review & editing: **F. L. N.**, **M. P.**, **C. M.**, **M. B.**, **R. S.**, **C. G.**, **G. T.**, **B. C**.

## FUNDING INFORMATION

This work was funded by the Deutsche Forschungsgemeinschaft (DFG, German Research Foundation)—178316478 C2 (F.L.N., M.P, C.M., M.B, R.S., C.G., G.T., B.C.).

## CONFLICT OF INTEREST STATEMENT

The authors declare no competing interests in relation to this study.

## Supporting information


**Appendix S1:** Supporting information.

## Data Availability

The data that support the findings of this study are available on request from the corresponding author. The data are not publicly available due to privacy or ethical restrictions.
