# Supplementary material for: Longitudinal microstructural alterations surrounding subcortical ischemic stroke lesions detected by free‐water imaging
Source: Hum Brain Mapp. 2024 May 23;45(8):e26722. doi: 10.1002/hbm.26722 (PMC11114091; doi:10.1002/hbm.26722)
Supplement: Supplementary file 1 — Appendix S1: Supporting information. [file HBM-45-e26722-s001.docx]

SUPPLEMENTARY MATERIAL

Longitudinal microstructural alterations surrounding subcortical ischemic stroke lesions detected by free-water imaging

Content

[Methods 2](#_Toc160706332)

[Study population 2](#_Toc160706333)

[Image acquisition 2](#_Toc160706334)

[Image processing 3](#_Toc160706335)

[Statistics 5](#_Toc160706336)

[Results 7](#_Toc160706337)

[Sample characteristics 7](#_Toc160706338)

[Imaging 8](#_Toc160706339)

[Sensitivity Analyses 12](#_Toc160706340)

[Complementary tract-of-interest analysis 25](#_Toc160706341)

[Power analysis 29](#_Toc160706342)

[References 30](#_Toc160706343)

# Methods

## Study population

**Figure S1**. Lesion distribution of the sample


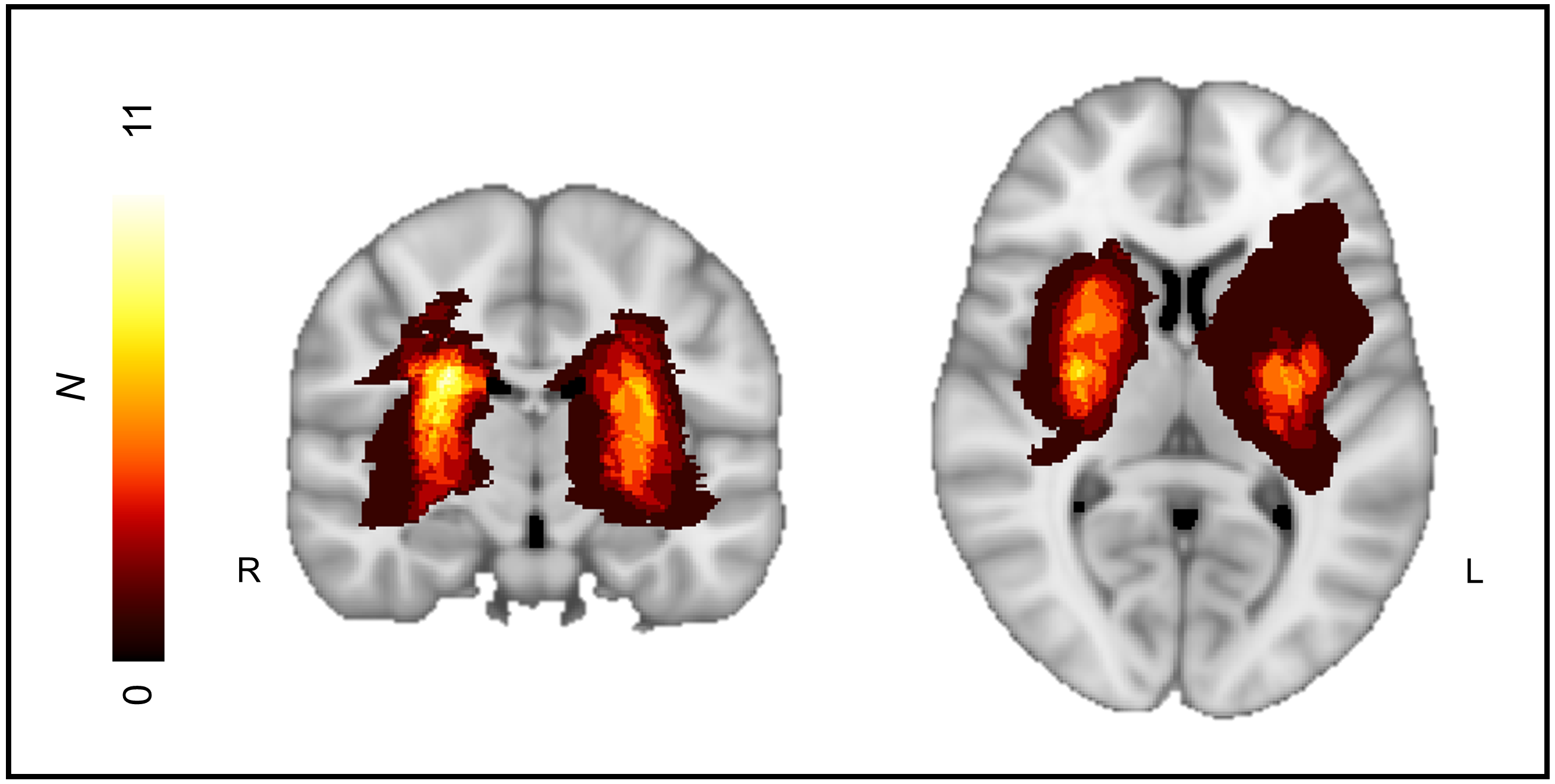


This figure shows a color coded overlay of all lesion masks on top of a T1-weighted MNI template in coronal (left) and axial (right) view. Images are shown in radiological convention.

## Image acquisition

Scanning was performed on a single 3T Siemens Skyra MRI scanner (Siemens, Er-langen, Germany) using a 32-channel head coil. Imaging sequences included high-resolution T1-weighted anatomical, FLAIR and diffusion weighted images (DWI). For T1-weighted MRI, a 3D magnetization-prepared rapid acquisition gradient-echo se-quence (MPRAGE) was employed with the following sequence parameters: repetition time (TR) = 2500 ms, echo time (TE) = 2.12 ms, field of view (FOV) = 240 x 192 mm, 256 axial slices, slice thickness (ST) = 0.94, in-plane resolution (IPR) = 0.94 x 0.94 mm. FLAIR images were acquired as follows: TR = 9000 ms, TE = 90 ms, TI = 2500 ms, FOV = 230 x 230 mm, ST = 5 mm, IPR = 0.7 x 0.7 mm. Finally, DWI included 75 axial slices with whole-brain coverage acquired along 64 non-collinear gradient directions (b = 1500 s/mm2) and one image with b = 0 s/mm2: TR = 10,000 ms, TE = 82 ms, FOV = 256 x 204, ST = 2 mm, IPR = 2 x 2 mm.

## Image processing

The following description is based on a boilerplate generated by *QSIPrep* and therefore facilitates standardized reporting and reproducibility.

*Anatomical data preprocessing*

We employed the longitudinal anatomical processing stream in order to ensure proper co-registration of individual time points for each participant. All T1w images were corrected for intensity non-uniformity (INU) using N4BiasFieldCorrection^1^ (ANTs 2.3.1). For each subject, a T1w-reference map was computed after registration of all T1w images/time points (after INU-correction) using mri_robust_template^2^ (FreeSurfer 6.0.1). The T1w-reference was then skull-stripped using antsBrainExtraction.sh (ANTs 2.3.1), using OASIS as target template. Brain surfaces were reconstructed using recon-all^3^ (FreeSurfer 6.0.1), and the brain mask estimated previously was refined with a custom variation of the method to reconcile ANTs-derived and FreeSurfer-derived segmentations of the cortical gray-matter of Mindboggle^4^.

*Diffusion data preprocessing*

MP-PCA denoising as implemented in MRtrix3’s dwidenoise^5^ was applied with a 5-voxel window. After MP-PCA, Gibbs unringing was performed using MRtrix3’s mrdegibbs^6^. Following unringing, B1 field inhomogeneity was corrected using dwibiascorrect from MRtrix3 with the N4 algorithm^1^.

*FSL*’s eddy (version 6.0.3:b862cdd5) was used for head motion correction and eddy current correction^7^. Eddy was configured with a *q*-space smoothing factor of 10, a total of 5 iterations, and 1000 voxels used to estimate hyperparameters. A linear first level model and a linear second level model were used to characterize eddy current-related spatial distortion. *q*-space coordinates were forcefully assigned to shells. Field offset was attempted to be separated from subject movement. Shells were aligned post-eddy. Eddy’s outlier replacement was run^8^. Data were grouped by slice, only including values from slices determined to contain at least 250 intracerebral voxels. Groups deviating by more than 4 standard deviations from the prediction had their data replaced with imputed values. Final interpolation was performed using the jac method.

A deformation field to correct for susceptibility distortions was estimated based on *fMRIPrep*’s^9^ fieldmap-less approach. The deformation field is that resulting from co-registering the b0 reference to the same-subject T1w-reference with its intensity inverted.^10,11^ Registration is performed with antsRegistration (ANTs 2.3.1), and the process regularized by constraining deformation to be nonzero only along the phase-encoding direction, and modulated with an average fieldmap template^12^. Based on the estimated susceptibility distortion, an unwarped b=0 reference was calculated for a more accurate co-registration with the anatomical reference. The DWI time-series were resampled to ACPC, generating a preprocessed DWI run in ACPC T1w space with 2 mm isotropic voxels.

Many internal operations of *QSIPrep* use *Nilearn* 0.8.0^13^ and *Dipy*^14^. For more details of the pipeline, see <https://qsiprep.readthedocs.io/en/latest/workflows.html>).

*Lesion analysis – quality assurance*

After the initial lesion masking, they were visually checked and manually edited by a research assistant to ensure accurate segmentations and coverage of visually apparent FLAIR and T1w signal alterations. Next, a second independent rater conducted visual quality control and editing. Deviations greater than 10% of the original lesion voxel size required a reconciliation with the person who prepared the initial lesion mask.

## Statistics

*Imaging – testing for differences between the lesion and tissue shells*

For each time point, we used linear mixed effects models with subject as random factor to test for differences in normalized free-water and fractional anisotropy (FA) of the tissue (FA_T_) between regions of interest adjusting for lesion volume and days from stroke to MRI session. Helmert contrasts were selected to test for the main effect *location of measurement*, thus comparing each level with the mean of the subsequent levels.

*Imaging – Complementary tract-of-interest analysis*

As a complementary analysis to support the interpretability and plausibility of alterations in imaging measures, we performed a tract-of-interest analysis, investigating longitudinal change in relative free-water and FA_T_ values of the ipsilateral corticospinal tract (CST), as well as linear regression analysis of CST tissue alterations with symptom severity (NIHSS) at 3 months after stroke.

As part of our standard free-water imaging pipeline (<https://github.com/csi-hamburg/CSIframe/blob/main/pipelines/freewater/freewater.sh>) we used antsRegistration^15^ to perform a non-linear registration of each subject’s FA map to the HCP1065 FA template in Montreal Neurological Institute (MNI) space available through the FMRIB software library^16^. The resulting transformation fields were subsequently applied to free-water and FA_T_ maps. Next, the HCP-1065 probabilistic white matter tract atlas derived by Yeh^17^ from imaging data of the Human Connectome Project^18^ was thresholded at 0.6 and consequently used to calculate mean free-water and FA_T_ for all 64 white matter tracts (left and right hemispheres separately). Finally, the ipsilesional diffusion markers were normalized to the contralesional, healthy side ([ipsilesional – contralesional] / contralesional). We focused the complimentary analysis on the corticospinal tract (CST) because it is predominantly affected in our sample of subcortical stroke patients.

We fitted longitudinal linear mixed effects models with *subject* as random intercept to test for differences in normalized free-water and FA_T_ of the CST across time points, adjusting for *lesion volume*, *age*, *sex,* and *days since stroke (z-scored within each time point)*. Dummy coding was used for contrasts, where the first time point (3-5 days after stroke) served as the reference, and Tukey post-hoc tests were performed for pairwise comparisons of time points.

We additionally tested for associations between free-water/FA_T_ in the CST and symptom severity as measured with the National Institutes of Health Stroke Scale (NIHSS) 3 months after stroke using Spearman correlations and linear models adjusting for *lesion volume*, *age*, *sex* and *days since stroke*. Moreover, utilizing Spearman correlations we explored potential links between free-water in the chronic stage, i.e. 1 year after stroke, and FA_T_ of the CST.

# Results

## Sample characteristics

**Figure S2**. Lesion volumes across time points


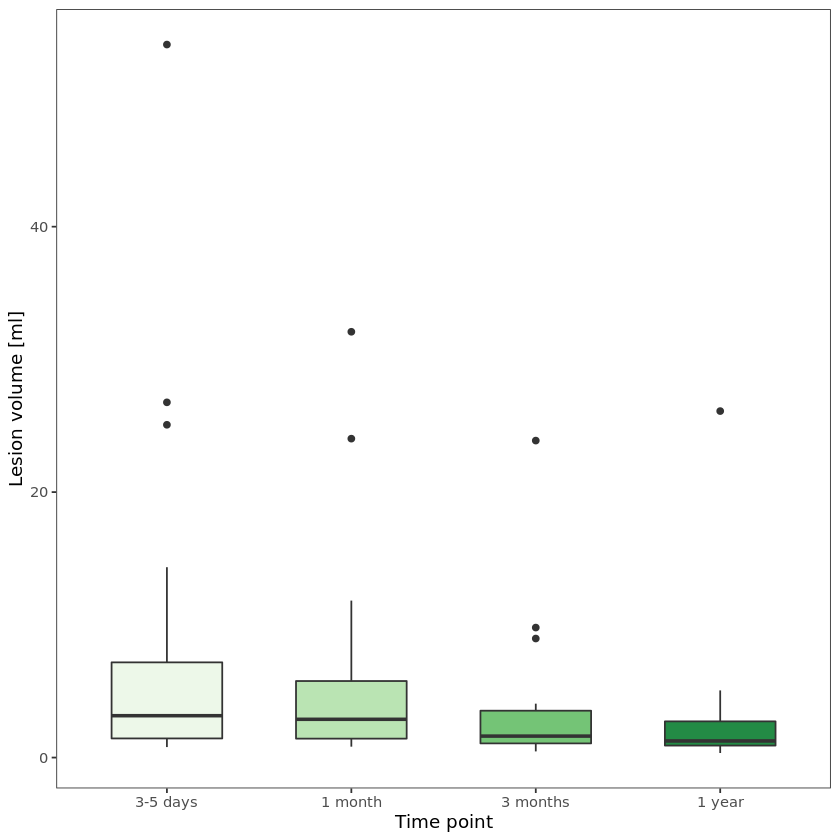


| **Table S1, Sample characteristics stratified by sex** | | | | |
| --- | --- | --- | --- | --- |
| **Variable^#^** | **Female (*N* = 11)** |  | **Male (*N* = 16)** | ***P*** |
| **Age in years** | 74.36 (10.42) |  | **61.38 (8.82)** | **.002**** |
| **Cardiovascular risk factors, count (%)**  **Arterial hypertension**  **Hypercholesterinemia**  **Diabetes**  **Smoking**  **Adipositas** | 9 (100)  4 (44.4)  2 (22.2)  3 (33.3)  3 (33.3) |  | 13 (86.7)  6 (40.0)  3 (20.0)  9 (60.0)  2 (13.3) | .70  >.99  >.99  .40  52 |
| **Lesion side, right, count (%)** | 7 (63.6) |  | 8 (50.0) | .76 |
| **Lesion volume in ml** | 11.26 (15.91) |  | 6.40 (8.10) | .31 |
| **NIHSS, median [IQR]** | 5 [2, 6] |  | 4 [3, 7] | >.99 |
| **Relative grip strength of affected hand** | 0.41 (0.50) |  | 0.47 (0.34) | .76 |
| **UEFM** | 37.88 (29.32) |  | 37.94 (26.18) | >.99 |
| **NHP** | 1.32 (0.17) |  | 2.49 (1.75) | .23 |
| **^#^**If not specified otherwise, data are presented as mean (standard deviation)  ******significant at *P*<.01  *Abbreviations:* IQR = inter-quartile range, NHP = Nine-Hole-Peg-Test, NIHSS = National Institutes of Health Stroke Scale, UEFM = Fugl-Meyer assessment of the upper extremity | | | | |

## Imaging

*Cross-sectional analyses comparing ipsilateral tissue properties between lesion and tissue shells*

Detailed results of the linear mixed effects models investigating differences in relative free-water and FA_T_ between ipsilateral lesion and tissue shells can be found in **Table S2**. Briefly, there was a significant main effect of *location of measurement* both in free-water and FA_T_ models at each time point (*P*<.001). Helmert contrasts revealed greater relative increases of free-water, as well as decreases of FA_T_ in the lesion and the 2 mm tissue shell compared to subsequent shells, respectively. One month after stroke, relative FA_T_ was additionally decreased at 4 mm distance comparing to shells with greater distance and there was a significant effect of *days since stroke* in the FA_T_ model (*β*=0.002, *P*=.02). Further, at 3 months and 1 year after stroke, *lesion volume* predicted lower relative FA_T_ values (*β*=-0.007, *P*=.002, *β*=-0.004, *P*=.04; respectively). Lastly, 1 year after stroke, older *age* and male *sex* was associated with lower relative free-water values (*β*=-0.021, *P*=.004, *β*=-0.417, *P*=.009; respectively.

| **Table S2. Results of the linear mixed effects models investigating differences in free-water and FA_T_ between lesion and tissue shells at four different time points** | | | | | | |
| --- | --- | --- | --- | --- | --- | --- |
|  | **Free-water** | | |  | **FA_T_** |  |
|  | **Estimate (SE)** | | ***P*** |  | **Estimate (SE)** | ***P*** |
| **Days 3-5 (N = 26)** | | | | | | |
| **Intercept** | -0.181 (0.330) | .59 | |  | -0.047 (0.069) | .51 |
| **Location** |  |  | |  |  |  |
| **Lesion** | 0.309 (0.041) | **<.001***** | |  | -0.325 (0.011) | **<.001***** |
| **2 mm** | 0.163 (0.041) | **<.001***** | |  | -0.058 (0.011) | **<.001***** |
| **4 mm** | 0.028 (0.041) | .50 | |  | 0.002 (0.011) | .88 |
| **6 mm** | 0.010 (0.042) | .82 | |  | 0.007 (0.011) | .49 |
| **8 mm** | 0.007 (0.043) | .88 | |  | 0.012 (0.011) | .28 |
| **10 mm** | -0.022 (0.044) | .62 | |  | 0.011 (0.011) | .33 |
| **12 mm** | -0.024 (0.047) | .60 | |  | 0.005 (0.012) | .66 |
| **14 mm** | -0.018 (0.054) | .74 | |  | -0.004 (0.014) | .78 |
| **Age** | 0.002 (0.004) | .55 | |  | <-0.001 (0.001) | .87 |
| **Sex (male)** | 0.041 (0.078) | .60 | |  | 0.028 (0.016) | .10 |
| **Lesion volume** | 0.002 (0.003) | .43 | |  | <-0.001 (0.001) | .84 |
| **Days since stroke** | 0.032 (0.034) | .36 | |  | -0.004 (0.007) | .57 |
| **1 month (N = 21)** | | | | | | |
| **Intercept** | 0.329 (0.306) | .30 | |  | -0.116 (0.055) | .05 |
| **Location** |  |  | |  |  |  |
| **Lesion** | 1.041 (0.069) | **<.001***** | |  | -0.318 (0.012) | **<.001***** |
| **2 mm** | 0.148 (0.070) | **.04*** | |  | -0.115 (0.012) | **<.001***** |
| **4 mm** | -.021 (0.071) | .77 | |  | -0.029 (0.013) | **.02*** |
| **6 mm** | 0.001 (0.072) | .99 | |  | -0.010 (0.013) | .45 |
| **8 mm** | -0.024 (0.073) | .75 | |  | -0.002 (0.013) | .88 |
| **10 mm** | -0.049 (0.076) | .52 | |  | -0.002 (0.013) | .87 |
| **12 mm** | -0.027 (0.080) | .73 | |  | -0.004 (0.014) | .79 |
| **14 mm** | -0.019 (0.093) | .84 | |  | -0.011 (0.016) | .49 |
| **Age** | -0.001 (0.004) | .87 | |  | -0.001 (0.001) | .33 |
| **Sex (male)** | -0.035 (0.081) | .67 | |  | 0.005 (0.015) | .76 |
| **Lesion volume** | 0.002 (0.005) | .65 | |  | -0.001 (0.001) | .20 |
| **Days since stroke** | -0.002 (0.004) | .56 | |  | 0.002 (0.001) | **.02*** |

| **Table S2. (continued)** | | | | | | |
| --- | --- | --- | --- | --- | --- | --- |
|  | **Free-water** | | |  | **FA_T_** |  |
|  | **Estimate (SE)** | | ***P*** |  | **Estimate (SE)** | ***P*** |
| **3 months (N = 19)** | | | | | | |
| **Intercept** | 0.602 (0.707) | .41 | |  | 0.069 (0.115) | **.**56 |
| **Location** |  |  | |  |  |  |
| **Lesion** | 1.943 (0.132) | **<.001***** | |  | -0.208 (0.016) | **<.001***** |
| **2 mm** | 0.344 (0.133) | **.01*** | |  | -0.119 (0.016) | **<.001***** |
| **4 mm** | 0.046 (0.135) | .73 | |  | -0.052 (0.016) | **.001**** |
| **6 mm** | 0.069 (0.137) | .62 | |  | -0.031 (0.016) | 0.05 |
| **8 mm** | 0.043 (0.139) | .76 | |  | -0.013 (0.017) | .42 |
| **10 mm** | -0.018 (0.144) | .90 | |  | <-0.001 (0.017) | .99 |
| **12 mm** | 0.008 (0.153) | .96 | |  | 0.005 (0.018) | .79 |
| **14 mm** | -0.028 (0.176) | .88 | |  | 0.003 (0.021) | .90 |
| **Age** | -0.005 (0.006) | .43 | |  | -0.002 (0.001) | .08 |
| **Sex (male)** | -0.091 (0.162) | .58 | |  | -0.006 (0.026) | .81 |
| **Lesion volume** | -0.007 (0.011) | .57 | |  | -0.007 (0.002) | **.002**** |
| **Days since stroke** | 0.002 (0.004) | .63 | |  | <0.001 (0.001) | .79 |
| **12 months (N = 19)** | | | | | | |
| **Intercept** | 5.033 (1.499) | **.005**** | |  | 0.125 (0.235) | .60 |
| **Location** |  |  | |  |  |  |
| **Lesion** | 2.174 (0.133) | **<.001***** | |  | -0.143 (0.018) | **<.001***** |
| **2 mm** | 0.862 (0.134) | **<.001***** | |  | -0.090 (0.018) | **<.001***** |
| **4 mm** | 0.247 (0.136) | .7 | |  | -0.029 (0.018) | .11 |
| **6 mm** | 0.169 (0.137) | .22 | |  | -0.021 (0.018) | .25 |
| **8 mm** | 0.102 (0.140) | .47 | |  | -0.012 (0.019) | .51 |
| **10 mm** | 0.048 (0.145) | .74 | |  | -0.004 (0.019) | .83 |
| **12 mm** | 0.060 (0.154) | .70 | |  | 0.006 (0.019) | .75 |
| **14 mm** | 0.039 (0.177) | .83 | |  | <0.001 (0.023) | >.99 |
| **Age** | -0.021 (0.006) | **.004**** | |  | -0.001 (<0.001) | .32 |
| **Sex (male)** | -0.417 (0.137) | **.009**** | |  | 0.014 (0.021) | .53 |
| **Lesion volume** | 0.001 (0.010) | .89 | |  | -0.004 (0.002) | **.04*** |
| **Days since stroke** | -0.008 (0.004) | .05 | |  | <0.001 (<0.001) | .58 |
| *Abbreviations*: FA_T_ = fractional anisotropy of the tissue, SE = standard error | | | | | | |

| **Table S3. Spearman correlations of imaging parameters 3-5 days after stroke with clinical variables and lesion volume** | | | | | | | | | | |
| --- | --- | --- | --- | --- | --- | --- | --- | --- | --- | --- |
|  | **Free-water** | | | | |  | **FA_T_** | | | |
| **Outcome variable** | | **Lesional** | | **Perilesional** | |  | **Lesional** | | **Perilesional** | |
|  | | ***Rho*** | ***P*** | ***Rho*** | ***P*** |  | ***Rho*** | ***P*** | ***Rho*** | ***P*** |
| **Baseline lesion volume (TP1), N = 26** | | 0.06 | .79 | 0.18 | .39 |  | 0.31 | .12 | -0.10 | .64 |
| **Change in lesion size (TP1-2), N = 20** | | 0.13 | .59 | -0.15 | 0.51 |  | -0.40 | .08 | -0.16 | .50 |
| **Change in lesion size (TP1-3), N = 18** | | 0.14 | .59 | **-0.51** | **.03*** |  | **-0.51** | **.03*** | -0.09 | .71 |
| **NIHSS (TP3) , N = 18** | | -0.29 | .24 | 0.18 | .47 |  | -0.15 | .55 | -0.08 | .74 |
| **Relative grip strength (TP3) , N = 17** | | 0.21 | .42 | -0.22 | .40 |  | -0.04 | .89 | -0.06 | .82 |
| **UEFM (TP3), N = 18** | | 0.18 | .47 | 0.05 | .85 |  | 0.19 | .46 | -0.15 | .56 |
| **NHP (TP3), N = 13** | | 0.45 | .12 | 0.49 | .09 |  | 0.02 | .96 | -0.22 | .47 |
| *Abbreviations*: FA_T_ = fractional anisotropy of the tissue*,* NHP = Nine-Hole-Peg-Test, NIHSS = National Institutes of Health Stroke Scale, SD = standard deviation, TP1 = 3-5 days after stroke, TP2 = 1 month after stroke, TP3 = 3 months after stroke, UEFM = Fugl-Meyer assessment of the upper extremity | | | | | | | | | | |

## Sensitivity Analyses

*Longitudinal linear mixed effect models for lesional and perilesional tissue separately*

We repeated the longitudinal linear mixed effects models for both imaging markers measured in the lesion and perilesional tissue separately. While the temporal trajectories of free-water and FA_T_ changes were very similar for both lesional and perilesional measures individually, compared to the combined model, it was evident that the amount of relative difference (ispilesionally vs. contralesionally) was much stronger in the lesion compared to the perilesional tissue (**Tables S4** and **S5**), in line with our supplementary cross-sectional models comparing each region of interest with the mean of more distant regions (**Table S2**).

| **Table S4. Results of the longitudinal linear mixed effects models investigating differences in free-water and FA_T_ as measured in the stroke lesion between different time points** | | | | | |
| --- | --- | --- | --- | --- | --- |
|  | **Free-water** | |  | **FA_T_** | |
| **R^2^ conditional / marginal*** | 0.554 / 0.472 | |  | 0.601 / 0.322 | |
|  | **Estimate (SE)** | ***P*** |  | **Estimate (SE)** | ***P*** |
| **Intercept^a^** | 4.357 (1.168) | **<.001***** |  | -0.401 (0.147) | **.01*** |
| **Lesion volume^a^** | -0.029 (0.015) | .06 |  | 0.002 (0.002) | .26 |
| **Age^a^** | -0.049 (0.015) | **0.003**** |  | 0.001 (0.002) | .73 |
| **Sex (male)^a^** | -0.808 (0.331) | **.02*** |  | 0.003 (0.042) | .95 |
| **Days since stroke, z-scored within time point^a^** | 0.007 (0.108) | .95 |  | 0.008 (0.010) | .40 |
| **Time point^a^** |  | **<.001***** |  |  | **<.001***** |
| **1 month – 3-5 days^b^** | 0.630 (0.282) | .11 |  | -0.028 (0.026) | .69 |
| **3 months – 3-5 days^b^** | 1.563 (0.296) | **<.001***** |  | 0.091 (0.028) | **.005**** |
| **1 year – 3-5 days^b^** | 1.971 (0.301) | **<.001***** |  | 0.171 (0.028) | **<.001***** |
| **3 months – 1 month^b^** | 0.934 (0.299) | **.01*** |  | 0.120 (0.027) | **<.001***** |
| **1 year – 1 month^b^** | 1.341 (0.301) | **<.001***** |  | 0.200 (0.027) | **<.001***** |
| **1 year – 3 months^b^** | 0.408 (0.305) | .54 |  | 0.080 (0.028) | **.02*** |
| ^a^Linear mixed-effects models with diffusion parameter as dependent variable, time point, lesion volume, age, sex and days since stroke (z-scored within time point) as fixed effects, and subject, as well as location of measurement as random intercept  ^b^Post-hoc Tukey’s tests comparing different time points with one another  *conditional: proportion of variance explained by both the fixed and random effects in the model; marginal: proportion of variance explained by the fixed effects in the model  *Abbreviations*: FA_T_ = fractional anisotropy of the tissue, SE = standard error | | | | | |

| **Table S5. Results of the longitudinal linear mixed effects models investigating differences in free-water and FA_T_ as measured in the perilesional tissue between different time points** | | | | | |
| --- | --- | --- | --- | --- | --- |
|  | **Free-water** | |  | **FA_T_** | |
| **R^2^ conditional / marginal*** | 0.461 / 0.123 | |  | 0.539 / 0.056 | |
|  | **Estimate (SE)** | ***P*** |  | **Estimate (SE)** | ***P*** |
| **Intercept^a^** | 0.378 (0.254) | .15 |  | -0.028 (0.066) | .68 |
| **Lesion volume^a^** | -0.004 (0.002) | .02 |  | <0.001 (<0.001) | .82 |
| **Age^a^** | -0.003 (0.003) | .40 |  | <0.001 (0.001) | .91 |
| **Sex (male)^a^** | -0.088 (0.074) | .25 |  | 0.020 (0.019) | .30 |
| **Days since stroke, z-scored within time point^a^** | -0.011 (0.010) | .30 |  | 0.001 (0.002) | .50 |
| **Time point^a^** |  | **<.001***** |  |  | **<.001***** |
| **1 month – 3-5 days^b^** | -0.038 (0.027) | .48 |  | -0.035 (0.006) | **<.001***** |
| **3 months – 3-5 days^b^** | 0.046 (0.029) | .38 |  | -0.039 (0.006) | **<.001***** |
| **1 year – 3-5 days^b^** | 0.241 (0.029) | **<.001***** |  | -0.027 (0.006) | **<.001***** |
| **3 months – 1 month^b^** | 0.085 (0.028) | **.01*** |  | -0.004 (0.006) | .89 |
| **1 year – 1 month^b^** | 0.279 (0.028) | **<.001***** |  | 0.007 (0.006) | .59 |
| **1 year – 3 months^b^** | 0.195 (0.028) | **<.001***** |  | 0.011 (0.006) | .20 |
| ^a^Linear mixed-effects models with diffusion parameter as dependent variable, time point, lesion volume, age, sex and days since stroke (z-scored within time point) as fixed effects, and subject, as well as location of measurement as random intercept  ^b^Post-hoc Tukey’s tests comparing different time points with one another  *conditional: proportion of variance explained by both the fixed and random effects in the model; marginal: proportion of variance explained by the fixed effects in the model  *Abbreviations*: FA_T_ = fractional anisotropy of the tissue, SE = standard error | | | | | |

**Figure S3**. Bar graphs visualizing relative change in free-water and FA_T_ by time point in the lesion and perilesional tissue


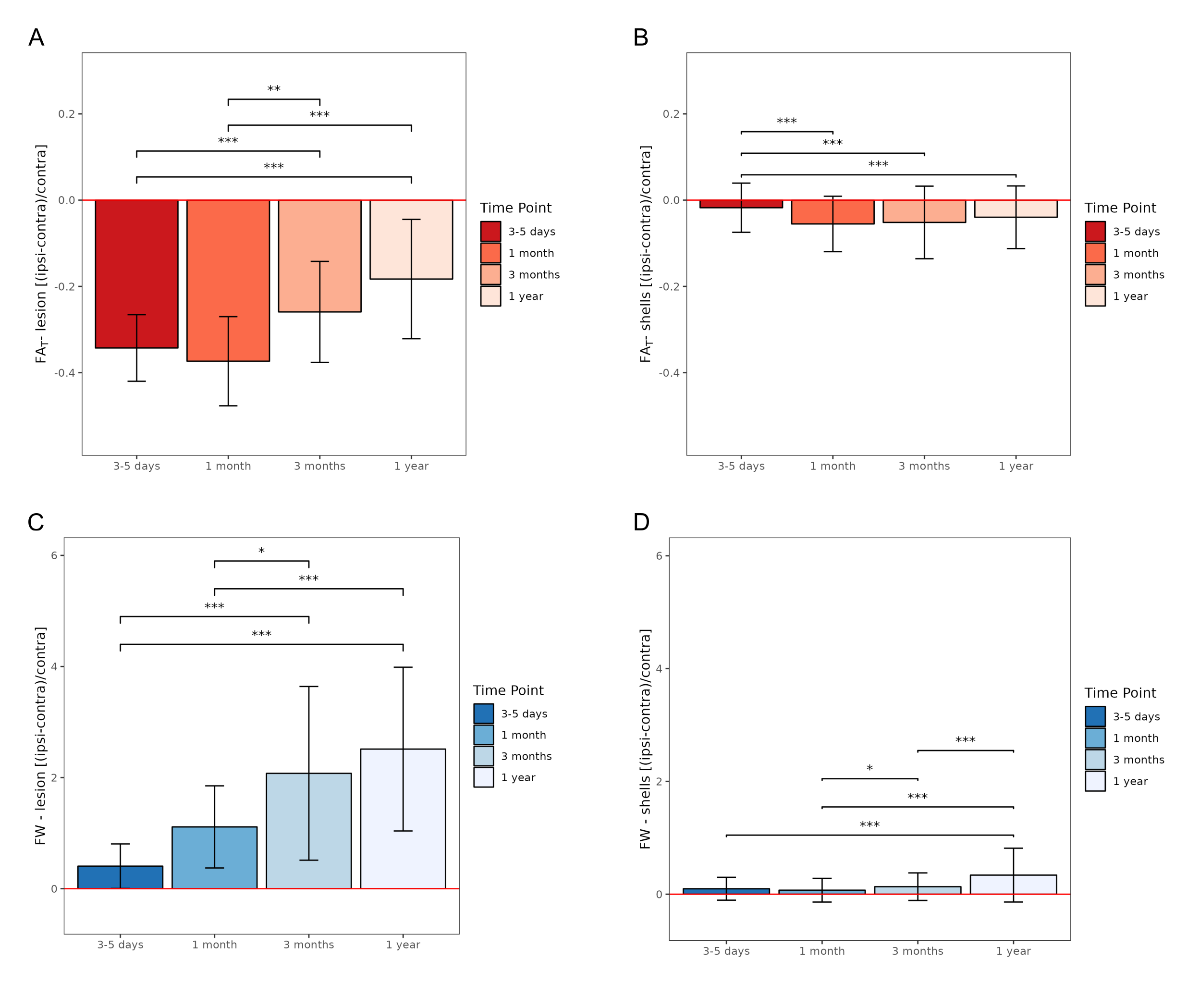


**Panel A and C** show the relative ipsilateral change in FA_T_ (A) and free-water (C) in the lesion for each time point. **Panel B** and **C** show the correspoding values for the perilesional tissue averaged across all tissue shells. The whiskers extend from one standard deviation above to one below the mean. Asterisks indicate the level of significance (****P*<.001, ***P*<.01, **P*<.05) derived from longitudinal linear mixed-effects models and post-hoc Tukey’s tests (**Tables S4 and S5**).

*Validation analysis in patients with supratentorial (cortical and subcortical) ischemic stroke*

The validation analysis was conducted on a combined sample of 39 patients with both cortical and subcortical stroke. Their clinical characteristics can be found in **Table S6**. In brief, the number of regions with significantly altered tissue microstructure detected by one-sample t-tests was slightly higher, although the relative differences between ipsi- and contralesional tissues were very similar (**Table S7** and **Figure S4**). The longitudinal linear mixed effects models showed almost identical results compared to the subcortical sample, besides that there were no significant *age* and *sex* effects on relative free-water (**Table S8**).

| **Table S6. Clinical characteristics of the validation sample** | | |
| --- | --- | --- |
| **Variable*** | **Patients with supratentorial ischemic stroke** | |
| **Age in years** | 65.92 (12.33), 39 | |
| **Female sex, count (%), N** | 17 (43.6 %), 39 | |
| **Lesion side, left, count (%), N** | 17 (43.6 %), 39 | |
| **Cardiovascular risk factors, count (%), N**  **Arterial hypertension**  **Hypercholesterinaemia**  **Diabetes**  **Smoking**  **Adipositas** | 32 (94.1), 34  15 (44.1), 34  5 (15.2), 33  16 (47.1), 34  8 (23.5), 34 | |
|  | **3-5 days after stroke** | **3 months after stroke** |
| **Lesion volume in ml**** | 13.60 (20.61), 37 | 8.39 (13.15), 27 |
| **NIHSS***** | 4 (2-7), 37 | 1 (0-3), 27 |
| **Relative grip strength of affected hand** | 0.49 (0.36), 34 | 0.75 (0.36), 26 |
| **UEFM** | 41.17 (24.73), 36 | 53.93 (20.39), 27 |
| **NHP** | 2.52 (1.95), 19 | 1.33 (0.34), 21 |
| *If not specified otherwise, data are presented as mean (standard deviation [SD]), N  **Of patients with available DWI data at the respective time point.  ***Presented as median (inter-quartile range)  *Abbreviations*: NHP = Nine-Hole-Peg-Test, NIHSS = National Institutes of Health Stroke Scale, SD = standard deviation, UEFM = Fugl-Meyer assessment of the upper extremity | | |

| **Table S7. Results of one-sample t-tests for each time point, investigating whether ipsilesional diffusion parameters are different compared to corresponding contralateral regions.** | | | | | | | |
| --- | --- | --- | --- | --- | --- | --- | --- |
| **Location** | **Relative free-water** | | |  | **Relative FA_T_** | | |
|  | **Mean % [95% CI]** | ***d**** | ***P*** |  | **Mean % [95% CI]** | ***d**** | ***P*** |
| **3-5 days (N = 37)** | | | | | | | |
| **Lesion** | 29.0 [14.2, 43.9] | 0.92 | **< .001***** |  | -35.9 [-38.3, -33.5] | -6.98 | **< .001***** |
| **2 mm** | 22.9 [13.0, 32.8] | 1.09 | **< .001***** |  | -7.4 [-9.8, -4.9] | -1.42 | **< .001***** |
| **4 mm** | 8.8 [0.01.2, 16.5] | 0.54 | **.03*** |  | -0.01.8 [-4.1, 0.4] | -0.39 | .10 |
| **6 mm** | 7.8 [1.8, 13.9] | 0.61 | **.01*** |  | -0.01.5 [-3.3, 0.2] | -0.42 | .08 |
| **8 mm** | 7.4 [1.8, 13.1] | 0.62 | **.01*** |  | -1.2 [-2.8, 0.4] | -0.35 | .15 |
| **10 mm** | 4.4 [-1.2, 10.0] | 0.37 | .12 |  | -1.2 [-2.9, 0.4] | -0.36 | .14 |
| **12 mm** | 4.4 [-1.4, 10.2] | 0.36 | .13 |  | -0.01.7 [-3.4, -0.1] | -0.49 | **.04*** |
| **14 mm** | 5.7 [-0.5, 11.8] | 0.43 | .07 |  | -0.02.4 [-3.7, -1.0] | -0.82 | **.001**** |
| **16 mm** | 7.2 [1.3, 13.1] | 0.57 | **.02*** |  | -0.02.3 [-3.5, -1.0] | -0.86 | **<.001***** |
| **1 month (N = 31)** | | | | | | | |
| **Lesion** | 102.0 [73.2, 130.9] | 1.83 | **<.001***** |  | -35.2 [-39.8, -30.6] | -3.98 | **< .001***** |
| **2 mm** | 21.2 [11.9, 30.5] | 1.18 | **< .001***** |  | -15.2 [-18.5, -11.9] | -2.39 | **< .001***** |
| **4 mm** | 7.3 [-1.2, 15.7] | 0.44 | .09 |  | -6.5 [-9.9, -3.1] | -0.99 | **< .001***** |
| **6 mm** | 9.5 [3.0, 15.9] | 0.76 | **.005**** |  | -5.4 [-7.7, -3.2] | -1.26 | **< .001***** |
| **8 mm** | 7.4 [1.0, 13.8] | 0.60 | **.02*** |  | -4.3 [-6.5, -2.1] | -1.02 | **< .001***** |
| **10 mm** | 6.2 [-0.4, 12.9] | 0.49 | .06 |  | -4.0 [-6.0, -2.0] | -1.05 | **< .001***** |
| **12 mm** | 7.2 [0.4, 14.1] | 0.55 | **.04*** |  | -4.1 [-6.0, -2.2] | -1.11 | **< .001***** |
| **14 mm** | 7.5 [-0.2, 15.1] | 0.51 | 0.06 |  | -3.9 [-5.6, -2.2] | -1.21 | **< .001***** |
| **16 mm** | 8.7 [0.2, 17.2] | 0.53 | **.05*** |  | -2.5 [-4.1, -0.9] | -0.80 | **.004**** |

| **Table S7. (continued)** | | | | | | | | |
| --- | --- | --- | --- | --- | --- | --- | --- | --- |
| **Location** | **Relative free-water** | | |  | **Relative FA_T_** | | | |
|  | **Mean % [95% CI]** | ***d**** | ***P*** |  | **Mean % [95% CI]** | | ***d**** | ***P*** |
| **3 months (N = 27)** | | | | | | | | |
| **Lesion** | 194.7 [138.8, 250.5] | 1.95 | **< .001***** |  | -24.7 [-29.1, -20.3] | -3.15 | | **< .001***** |
| **2 mm** | 49.3 [34.6, 63.9] | 1.88 | **< .001***** |  | -15.7 [-19.1, -12.3] | -2.61 | | **< .001***** |
| **4 mm** | 22.9 [12.1, 33.8] | 1.18 | **< .001***** |  | -8.4 [-12.2, -4.7] | -1.25 | | **< .001***** |
| **6 mm** | 21.8 [12.5, 31.1] | 1.31 | **< .001***** |  | -5.5 [-8.7, -2.3] | -0.97 | | **.001**** |
| **8 mm** | 19.3 [10.9, 27.7] | 1.29 | **< .001***** |  | -4.1 [-6.7, -1.5] | -0.87 | | **.004**** |
| **10 mm** | 12.4 [5.1, 19.7] | 0.95 | **.002**** |  | -3.2 [-5.7, -0.7] | -0.71 | | **.02*** |
| **12 mm** | 12.5 [5.0, 20.0] | 0.93 | **.002**** |  | -2.5 [-4.8, -0.1] | -0.59 | | **.04*** |
| **14 mm** | 8.1 [1.1, 15.1] | 0.65 | **.02*** |  | -2.3 [-4.4, -0.1] | -0.58 | | **.04*** |
| **16 mm** | 9.5 [1.3, 17.8] | 0.65 | **.03*** |  | -2.7 [-4.8, -0.7] | -0.74 | | **.01*** |
| **12 months (N = 26)** | | | | | | | | |
| **Lesion** | 239.4 [182.5, 296.4] | 2.40 | **<.001***** |  | -17.3 [-22.2, -12.3] | -1.99 | | **< .001***** |
| **2 mm** | 108.0 [78.7, 137.2] | 2.11 | **<.001***** |  | -9.9 [-15.7, -4.0] | -0.96 | | **.002**** |
| **4 mm** | 49.9 [33.3, 66.5] | 1.72 | **<.001***** |  | -5.0 [-8.6, -1.5] | -0.81 | | **.008**** |
| **6 mm** | 40.3 [28.0, 52.5] | 1.88 | **<.001***** |  | -4.8 [-7.2, -2.5] | -1.18 | | **< .001***** |
| **8 mm** | 32.9 [21.5, 44.4] | 1.64 | **<.001***** |  | -4.3 [-6.7, -1.9] | -1.01 | | **.001**** |
| **10 mm** | 25.8 [14.7, 36.8] | 1.33 | **<.001***** |  | -3.0 [-5.1, -1.0] | -0.84 | | **.006**** |
| **12 mm** | 23.6 [13.8, 33.4] | 1.38 | **<.001***** |  | -2.0 [-4.3, 0.4] | -0.48 | | .09 |
| **14 mm** | 19.2 [10.0, 28.3] | 1.20 | **<.001***** |  | -2.3 [-4.5, -0.2] | -0.61 | | **0.04*** |
| **16 mm** | 14.0 [5.8, 22.2] | 0.98 | **0.002**** |  | -2.6 [-4.6, -0.6] | -0.76 | | **0.01*** |
| *Abbreviations*: CI = confidence interval, FA_T_ = fractional anisotropy of the tissue  *Cohen’s d | | | | | | | | |

| **Table S8. Results of the longitudinal linear mixed effects models of the validation sample (including cortical infarcts) investigating differences in free-water and FA_T_ between different time points** | | | | | |
| --- | --- | --- | --- | --- | --- |
|  | **Free-water** | |  | **FA_T_** | |
| **R^2^ conditional / marginal*** | 0.506 / 0.093 | |  | 0.654 / 0.017 | |
|  | **Estimate (SE)** | ***P*** |  | **Estimate (SE)** | ***P*** |
| **Intercept^a^** | 0.462 (0.251) | .07 |  | -0.013 (0.049) | .80 |
| **Lesion volume^a^** | -0.002 (0.002) | .27 |  | <-0.001 (  <0.001) | .25 |
| **Age^a^** | -0.004 (0.003) | .14 |  | -0.001 (0.001) | .17 |
| **Sex (male)^a^** | -0.079 (0.068) | .25 |  | 0.001 (0.013) | .92 |
| **Days since stroke, z-scored within time point^a^** | 0.005 (0.016) | .74 |  | 0.003 (0.002) | .27 |
| **Time point^a^** |  | **<.001***** |  |  | **<.001***** |
| **1 month – 3-5 days^b^** | 0.075 (0.040) | .24 |  | -0.028 (0.006) | **<.001***** |
| **3 months – 3-5 days^b^** | 0.267 (0.043) | **<.001***** |  | -0.017 (0.006) | **.04*** |
| **1 year – 3-5 days^b^** | 0.491 (0.044) | **<.001***** |  | 0.002 (0.007) | .99 |
| **3 months – 1 month^b^** | 0.192 (0.041) | **<.001***** |  | 0.011 (0.006) | .29 |
| **1 year – 1 month^b^** | 0.416 (0.042) | **<.001***** |  | 0.030 (0.006) | **<.001***** |
| **1 year – 3 months^b^** | 0.225 (0.043) | **<.001***** |  | 0.019 (0.006) | **.01*** |
| ^a^Linear mixed-effects models with diffusion parameter as dependent variable, time point, lesion volume, age, sex and days since stroke (z-scored within time point) as fixed effects, and subject, as well as location of measurement as random intercept  ^b^Post-hoc Tukey’s tests comparing different time points with one another  *conditional: proportion of variance explained by both the fixed and random effects in the model; marginal: proportion of variance explained by the fixed effects in the model  *Abbreviations*: FA_T_ = fractional anisotropy of the tissue, SE = standard error | | | | | |

**Figure S4**. Box plots and bar graphs visualizing relative change in free-water and FA_T_ by time point and location of measurement in the validation sample


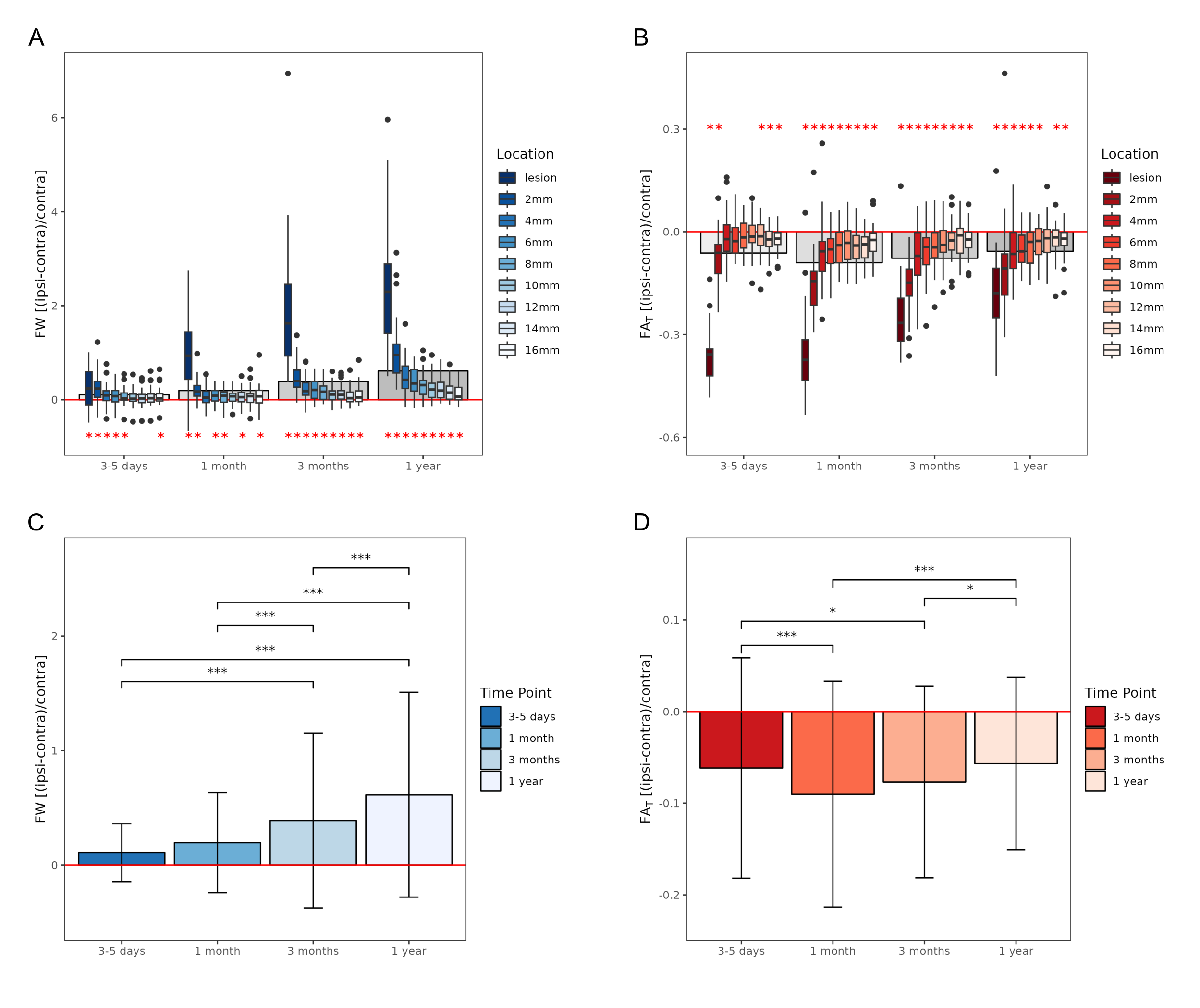


**Panel A and B** show the relative ispilateral change in free-water (A) and FA_T_ (B) compared to contralateral corresponding regions. The box plots are stratified by location of measurement and time point. The lower and upper end of the box corresonds to the 1^st^ and 3^rd^ quartiles, respectively. The line within the box represents the median. The whiskers extend to 1.5 times the interquartile range below and above the 1^st^ and 3^rd^ quartiles, respectively. The red horizontel line indicates the level of zero change, the gray rectangles in the background represent the mean across all regions of interest for each time point. Red asterisks indicate a significant difference from zero change (*P*<.05) as determined by one-sample t-tests for each region of interest, separately (**Table S7**).

**Panel C and D** show the relative ipsilateral change in free-water (C) and FA_T_ (D) averaged across regions of interest for each time point. The whiskers extend from one standard deviation above to one below the mean. Asterisks indicate the level of significance (****P*<.001, ***P*<.01, **P*<.05) derived from longitudinal linear mixed-effects models and post-hoc Tukey’s tests (**Table S8**).

| **Table S9. Results of the linear mixed effects models for patients with supratentorial ischemic stroke, investigating differences in free-water and FA_T_ between lesion and tissue shells at four different time points** | | | | | | |
| --- | --- | --- | --- | --- | --- | --- |
|  | **Free-water** | | |  | **FA_T_** |  |
|  | **Estimate (SE)** | | ***P*** |  | **Estimate (SE)** | ***P*** |
| **Days 3-5 (N = 37)** | | | | | | |
| **Intercept** | 0.225 (0.209) | .29 | |  | 0.014 (0.036) | .70 |
| **Location** |  |  | |  |  |  |
| **Lesion** | 0.205 (0.034) | **<.001***** | |  | -0.335 (0.008) | **<.001***** |
| **2 mm** | 0.163 (0.034) | **<.001***** | |  | -0.056 (0.008) | **<.001***** |
| **4 mm** | 0.027 (0.034) | .44 | |  | -0.001 (0.008) | .89 |
| **6 mm** | 0.020 (0.035) | .56 | |  | 0.002 (0.009) | .81 |
| **8 mm** | 0.020 (0.035) | .57 | |  | 0.007 (0.009) | .41 |
| **10 mm** | -0.014 (0.037) | .71 | |  | 0.009 (0.009) | .33 |
| **12 mm** | -0.020 (0.039) | .61 | |  | 0.006 (0.010) | .56 |
| **14 mm** | -0.015 (0.045) | .73 | |  | -0.001 (0.011) | .92 |
| **Age** | -0.002 (0.003) | .47 | |  | -0.001 (<.001) | .14 |
| **Sex (male)** | 0.008 (0.063) | .91 | |  | 0.008 (0.011) | .47 |
| **Lesion volume** | 0.001 (0.001) | .70 | |  | -0.001 (<.001) | **.006**** |
| **Days since stroke** | 0.001 (0.016) | .96 | |  | -0.005 (0.003) | .07 |
| **1 month (N = 31)** | | | | | | |
| **Intercept** | 0.345 (0.249) | 0.18 | |  | -0.027 (0.054) | .63 |
| **Location** |  |  | |  |  |  |
| **Lesion** | 0.926 (0.056) | **<.001***** | |  | -0.295 (0.012) | **<.001***** |
| **2 mm** | 0.135 (0.057) | **.02*** | |  | -0.108 (0.012) | **<.001***** |
| **4 mm** | -0.005 (0.057) | .93 | |  | -0.025 (0.012) | **.04*** |
| **6 mm** | 0.021 (0.058) | .72 | |  | -0.017 (0.012) | .17 |
| **8 mm** | <.001 (0.059) | >.99 | |  | -0.007 (0.013) | .59 |
| **10 mm** | -0.016 (0.061) | .80 | |  | -0.005 (0.013) | .70 |
| **12 mm** | -0.008 (0.065) | .90 | |  | -0.009 (0.014) | .53 |
| **14 mm** | -0.012 (0.075) | .87 | |  | -0.014 (0.016) | .38 |
| **Age** | -0.001 (0.003) | .70 | |  | -0.001 (0.001) | .05 |
| **Sex (male)** | -0.048 (0.072) | .51 | |  | -0.004 (0.016) | .81 |
| **Lesion volume** | 0.003 (0.003) | .24 | |  | -0.002 (0.001) | **.002**** |
| **Days since stroke** | -0.002 (0.004) | .65 | |  | 0.001 (0.001) | .20 |

| **Table S9. (continued)** | | | | | | |
| --- | --- | --- | --- | --- | --- | --- |
|  | **Free-water** | | |  | **FA_T_** |  |
|  | **Estimate (SE)** | | ***P*** |  | **Estimate (SE)** | ***P*** |
| **3 months (N = 27)** | | | | | | |
| **Intercept** | 0.351 (0.492) | .48 | |  | 0.034 (0.100) | .74 |
| **Location** |  |  | |  |  |  |
| **Lesion** | 1.752 (0.101) | **<.001***** | |  | -0.191 (0.012) | **<.001***** |
| **2 mm** | 0.341 (0.102) | **<.001***** | |  | -0.116 (0.012) | **<.001***** |
| **4 mm** | 0.090 (0.103) | .38 | |  | -0.051 (0.012) | **<.001***** |
| **6 mm** | 0.094 (0.104) | .37 | |  | -0.026 (0.012) | **.04*** |
| **8 mm** | 0.087 (0.106) | .41 | |  | -0.015 (0.013) | .25 |
| **10 mm** | 0.024 (0.110) | .83 | |  | -0.007 (0.013) | .61 |
| **12 mm** | 0.036 (0.117) | .76 | |  | <.001 (0.014) | .97 |
| **14 mm** | -0.014 (0.135) | .92 | |  | 0.005 (0.016) | .76 |
| **Age** | -0.001 (0.005) | .79 | |  | -0.002 (0.001) | .11 |
| **Sex (male)** | -0.065 (0.112) | .57 | |  | -0.005 (0.023) | .82 |
| **Lesion volume** | <0.001 (0.004) | .90 | |  | -0.002 (0.001) | **.04*** |
| **Days since stroke** | 0.002 (0.004) | .63 | |  | <.001 (0.001) | .97 |
| **12 months (N = 26)** | | | | | | |
| **Intercept** | 1.263 (1.566) | .43 | |  | 0.041 (0.179) | .82 |
| **Location** |  |  | |  |  |  |
| **Lesion** | 2.000 (0.107) | **<.001***** | |  | -0130 (0.016) | **<.001***** |
| **2 mm** | 0.786 (0.108) | **<.001***** | |  | -0.064 (0.016) | **<.001***** |
| **4 mm** | 0.238 (0.109) | **.03*** | |  | -0.018 (0.016) | .26 |
| **6 mm** | 0.172 (0.110) | .12 | |  | -0.020 (0.016) | .23 |
| **8 mm** | 0.123 (0.113) | .28 | |  | -0.018 (0.017) | .28 |
| **10 mm** | 0.068 (0.116) | .56 | |  | -0.007 (0.017) | .68 |
| **12 mm** | 0.070 (0.123) | .57 | |  | 0.005 (0.018) | .80 |
| **14 mm** | 0.051 (0.142) | .72 | |  | 0.003 (0.021) | .89 |
| **Age** | -0.006 (0.006) | .35 | |  | -0.001 (0.001) | .25 |
| **Sex (male)** | -0.191 (0.148) | .21 | |  | 0.011 (0.017) | .53 |
| **Lesion volume** | 0.007 (0.007) | .34 | |  | -0.002 (0.001) | **.02*** |
| **Days since stroke** | -0.001 (0.004) | .89 | |  | <.001 (<.001) | .82 |
| *Abbreviations*: SE = standard error | | | | | | |

| **Table S10. Spearman correlations of imaging parameters 3-5 days after supratentorial stroke with clinical variables and lesion volume** | | | | | | | | | | |
| --- | --- | --- | --- | --- | --- | --- | --- | --- | --- | --- |
|  | **Free-water** | | | | |  | **FA_T_** | | | |
| **Outcome variable** | | **Lesional** | | **Perilesional** | |  | **Lesional** | | **Perilesional** | |
|  | | ***Rho*** | ***P*** | ***Rho*** | ***P*** |  | ***Rho*** | ***P*** | ***Rho*** | ***P*** |
| **Baseline lesion volume (TP1), N = 37** | | 0.01 | .93 | 0.14 | .40 |  | 0.044 | .79 | -0.29 | .08 |
| **Change in lesion size (TP1-2), N = 29** | | 0.08 | .68 | -0.10 | .60 |  | -0.25 | 0.19 | 0.14 | .46 |
| **Change in lesion size (TP1-3), N = 25** | | -0.11 | .59 | **-0.41** | **.04** |  | **-0.41** | **.04** | 0.03 | .90 |
| **NIHSS (TP3) , N = 25** | | -0.24 | .25 | 0.08 | .72 |  | -0.26 | .21 | -0.32 | .12 |
| **Relative grip strength (TP3) , N = 24** | | 0.16 | .45 | 0.05 | .81 |  | -0.08 | .73 | 0.02 | .93 |
| **UEFM (TP3), N = 25** | | -0.10 | .65 | 0.28 | .18 |  | 0.11 | .62 | -0.05 | .80 |
| **NHP (TP3), N = 19** | | 0.16 | .52 | 0.45 | .05 |  | -0.03 | .89 | -0.34 | .15 |
| *Abbreviations*: FA_T_ = fractional anisotropy of the tissue*,* NHP = Nine-Hole-Peg-Test, NIHSS = National Institutes of Health Stroke Scale, SD = standard deviation, TP1 = 3-5 days after stroke, TP2 = 1 month after stroke, TP3 = 3 months after stroke, UEFM = Fugl-Meyer assessment of the upper extremity | | | | | | | | | | |

## Complementary tract-of-interest analysis

The longitudinal change in in imaging markers observed in our complimentary tract-of-interest analysis of the CST, was very similar to what was found in the tissue shell based analysis. FA_T_ decreases were again most prominent at 1 month after stroke followed by a gradual increase. Free-water steadily increased accentuated 1 month after stroke (**Table S11**, **Figure S5**). In contrast to the main analysis, relative FA_T_ of the CST was significantly associated with NIHSS 3 months after stroke (**Table S11**, **Figure S6**).

*Longitudinal linear mixed effects model for diffusion markers in the corticospinal tract*

| **Table S11. Results of the longitudinal linear mixed effects model investigating differences in free-water and FA_T_ as measured in the corticospinal tract between different time points** | | | | | |
| --- | --- | --- | --- | --- | --- |
|  | **Free-water** | |  | **FA_T_** | |
| **R^2^ conditional / marginal*** | 0.823 / 0.117 | |  | 0.850 / 0.219 | |
|  | **Estimate (SE)** | ***P*** |  | **Estimate (SE)** | ***P*** |
| **Intercept^a^** | 0.106 (0.250) | .67 |  | -0.146 (0.091) | .12 |
| **Lesion volume^a^** | 0.002 (0.002) | .30 |  | <-0.001 (0.001) | .79 |
| **Age^a^** | -0.001 (0.003) | .74 |  | 0.002 (0.001) | .20 |
| **Sex (male)^a^** | -0.008 (0.074) | .92 |  | 0.013 (0.027) | .64 |
| **Days since stroke, z-scored within time point^a^** | 0.008 (0.009) | .39 |  | -0.001 (0.003) | .71 |
| **Time point^a^** |  | **<.001***** |  |  | **<.001***** |
| **1 month – 3-5 days^b^** | 0.007 (0.023) | .99 |  | -0.063 (0.008) | **<.001***** |
| **3 months – 3-5 days^b^** | 0.081 (0.025) | **.006**** |  | -0.059 (0.009) | **<.001***** |
| **1 year – 3-5 days^b^** | 0.142 (0.025) | **<.001***** |  | -0.036 (0.009) | **<.001***** |
| **3 months – 1 month^b^** | 0.074 (0.024) | **.01*** |  | 0.004 (0.009) | .96 |
| **1 year – 1 month^b^** | 0.135 (0.024) | **<.001***** |  | 0.027 (0.009) | **.01**** |
| **1 year – 3 months^b^** | 0.061 (0.024) | .05 |  | 0.023 (0.009) | **.04*** |
| ^a^Linear mixed-effects models with diffusion parameter as dependent variable, time point, lesion volume, age, sex and days since stroke (z-scored within time point) as fixed effects, and subject, as well as location of measurement as random intercept  ^b^Post-hoc Tukey’s tests comparing different time points with one another  *conditional: proportion of variance explained by both the fixed and random effects in the model; marginal: proportion of variance explained by the fixed effects in the model  *Abbreviations*: FA_T_ = fractional anisotropy of the tissue, SE = standard error | | | | | |

**Figure S5.** Bar graphs visualizing relative change in free-water and FA_T_ by time point in the corticospinal tract


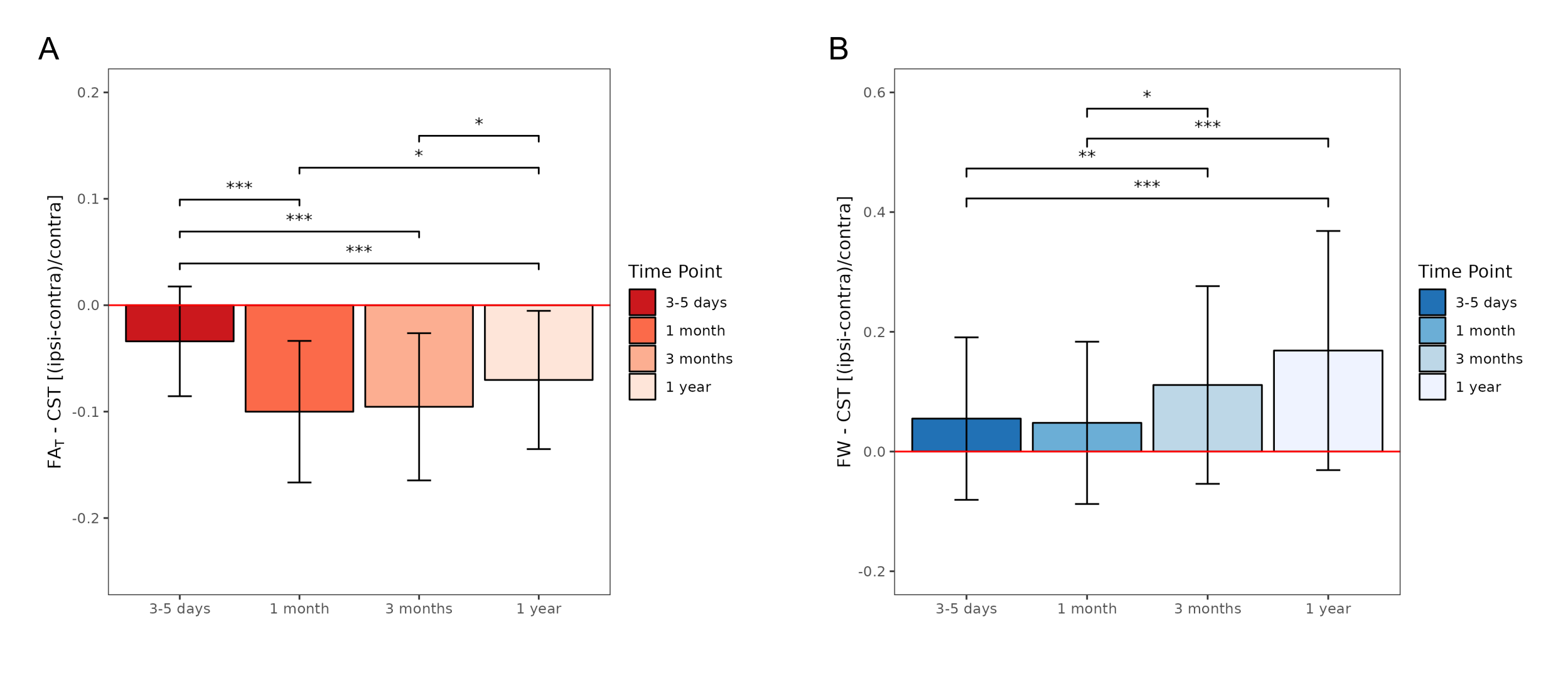


**Panel A and B** show the relative ipsilateral change in FA_T_ (A) and free-water (B) in the corticospinal tract (CST) for each time point.. The whiskers extend from one standard deviation above to one below the mean. Asterisks indicate the level of significance (****P*<.001, ***P*<.01, **P*<.05) derived from longitudinal linear mixed-effects models and post-hoc Tukey’s tests (**Table S11**).

*Associations of free-water and FA_T_ in the corticospinal tract 3 months after stroke with NIHSS*

**Figure S6**. Scatter plots and regression lines visualizing associations between relative free-water and FA_T_ with NIHSS 3 months after stroke


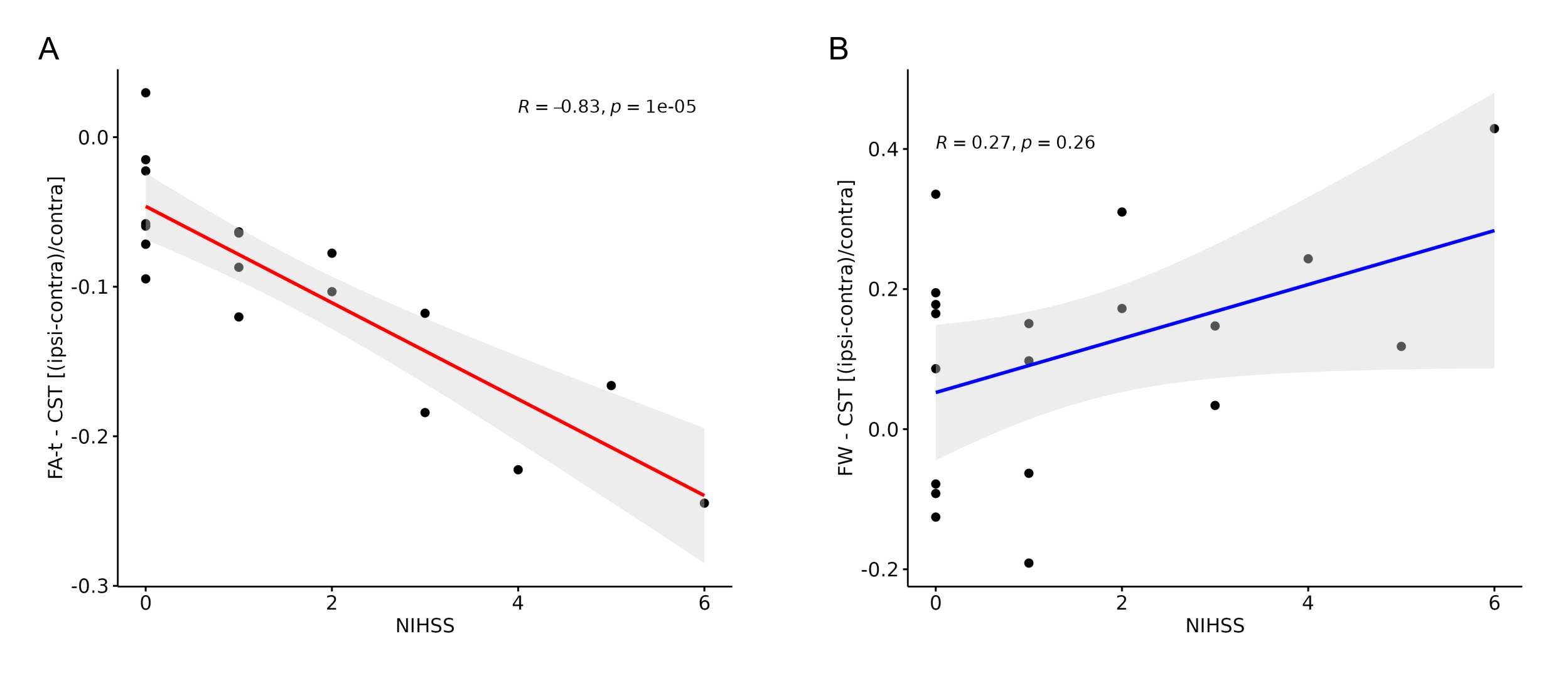


*Note: R* corresponds to Spearman’s *rho*

*Abbreviations*: CST = corticospinal tract, FA-t = fractional anisotropy of the tissue, FW = free-water, NIHSS = National Institutes of Health Stroke Scale

| **Table S12. Results of the linear models relating free-water and FA_T_ as measured in the corticospinal tract to NIHSS 3 months after stroke** | | | | | |
| --- | --- | --- | --- | --- | --- |
|  | **Free-water** | |  | **FA_T_** | |
| **Adjusted R^2^** | **0.375*** | |  | **0.740***** | |
|  | **Estimate (SE)** | ***P*** |  | **Estimate (SE)** | ***P*** |
| **Intercept** | 1.055 (4.267) | .81 |  | 1.620 (2.754) | .57 |
| **Diffusion parameter** | 3.698 (2.700) | .19 |  | -20.303 (4.262) | **<.001***** |
| **Age** | -0.071 (0.041) | .11 |  | -0.046 (0.027) | .11 |
| **Sex (male)** | 0.750 (0.980) | .46 |  | -0.056 (0.654) | .93 |
| **Days since stroke** | 0.038 (0.024) | 0.14 |  | 0.011 (0.016) | .50 |
| **Lesion volume** | 0.027 (0.082) | .74 |  | -0.054 (0.045) | .25 |
| **P-value of overall model < .05*  **** P-value of overall model <.001*  *Abbreviations*: FA_T_ = fractional anisotropy of the tissue, SE = standard error | | | | | |

*Associations of free-water with FA_T_ in the corticospinal tract 1 year after stroke*

| **Table S13. Spearman correlations of relative free-water 1 year after stroke with FA_T_ of the corticospinal tract** | | |
| --- | --- | --- |
|  | **FA_T_** | |
|  | ***Rho*** | ***P*** |
| **Lesional free-water** | -0.21 | .38 |
| **Perilesional free-water** | 0.09 | .70 |
| **CST free-water** | **-0.45** | .05 |
| *Abbreviations*: FA_T_ = fractional anisotropy of the tissue | | |

## Power analysis

In summary, the average post-hoc power of detecting differences between ispilesional and contralesional regions of interest for free-water and FA_T_ was 80% and 77%, respectively. The average power for detecting differences in diffusion markers between individual time points was 88% (range: 42-100%) for free-water and 55% (range 11%-99%) for FA_T_. While these results highlight a relatively high power and therefore low type II error probability for our study contrasts (albeit to a lower degree for FA_T_ especially in the longitudinal model), we would like to point out that such post-hoc power analyses should be interpreted with caution (Zhang et al. 2019, General Psychiatry).

| **Table S14. Post-hoc power analysis for the main study contrasts** | | | | | |
| --- | --- | --- | --- | --- | --- |
|  | **Free-water** | |  | **FA_T_** | |
| **Contrast** | ***Cohen’s d*** | ***Power*** |  | ***Cohen’s d*** | ***Power*** |
| **Time**  **3-5 days vs. 1 month**  **3-5 days vs. 3 months**  **3-5 days vs. 1 year**  **1 month vs 3 months**  **1 month vs. 1 year**  **3 months vs 1 year** | -1.17  -0.41  -1.30  -1.84  -1.07  -1.65  -0.78 | **0.88**  **0.42**  **>0.99**  **>0.99**  **0.99**  **>.99**  **0.88** |  | 0.13  1.21  0.68  0.26  -0.18  -0.80  -0.37 | **0.55**  **>0.99**  **0.77**  **0.18**  **0.11**  **0.89**  **0.32** |
| **Ipsilesional vs. contralesional**  **3-5 days**  **1 month**  **3 months**  **1 year** | 0.97  0.76  0.65  0.93  1.53 | **0.80**  **0.86**  **0.54**  **0.81**  **0.99** |  | -1.28  -1.11  -1.91  -1.19  -0.91 | **0.77**  **0.53**  **>0.99**  **0.79**  **0.75** |
| *Abbreviations*: FA_T_ = fractional anisotropy of the tissue  For each contrast the first line represents the average Cohen’s d and power across subcontrast listed thereafter. For the contrast *time*, relative differences in free-water and FA_T_ between ipsilesional and contralesional tissue was averaged across all regions of interest. For the contrast *ipsilesional vs. contralesional*, first, power and Cohen’s d were calculated for individual regions of interest and for each time point separately, before being averaged within each time point. | | | | | |

# References

1 Tustison NJ, Avants BB, Cook PA, Zheng Y, Egan A, Yushkevich PA *et al.* N4ITK: improved N3 bias correction. *IEEE Trans Med Imaging* 2010; **29**: 1310–1320.

2 Reuter M, Rosas HD, Fischl B. Highly accurate inverse consistent registration: A robust approach. *NeuroImage* 2010; **53**: 1181–1196.

3 Dale AM, Fischl B, Sereno MI. Cortical Surface-Based Analysis: I. Segmentation and Surface Reconstruction. *NeuroImage* 1999; **9**: 179–194.

4 Klein A, Ghosh SS, Bao FS, Giard J, Häme Y, Stavsky E *et al.* Mindboggling morphometry of human brains. *PLOS Comput Biol* 2017; **13**: e1005350.

5 Veraart J, Novikov DS, Christiaens D, Ades-aron B, Sijbers J, Fieremans E. Denoising of diffusion MRI using random matrix theory. *NeuroImage* 2016; **142**: 394–406.

6 Kellner E, Dhital B, Kiselev VG, Reisert M. Gibbs-ringing artifact removal based on local subvoxel-shifts. *Magn Reson Med* 2016; **76**: 1574–1581.

7 Andersson JLR, Sotiropoulos SN. An integrated approach to correction for off-resonance effects and subject movement in diffusion MR imaging. *NeuroImage* 2016; **125**: 1063–1078.

8 Andersson JLR, Graham MS, Zsoldos E, Sotiropoulos SN. Incorporating outlier detection and replacement into a non-parametric framework for movement and distortion correction of diffusion MR images. *NeuroImage* 2016; **141**: 556–572.

9 Esteban O, Markiewicz CJ, Blair RW, Moodie CA, Isik AI, Erramuzpe A *et al.* fMRIPrep: a robust preprocessing pipeline for functional MRI. *Nat Methods* 2019; **16**: 111–116.

10 Wang S, Peterson DJ, Gatenby JC, Li W, Grabowski TJ, Madhyastha TM. Evaluation of Field Map and Nonlinear Registration Methods for Correction of Susceptibility Artifacts in Diffusion MRI. *Front Neuroinformatics* 2017; **11**.https://www.frontiersin.org/article/10.3389/fninf.2017.00017 (accessed 16 May2022).

11 Huntenburg JM. *Evaluating nonlinear coregistration of BOLD EPI and T1w images*. 2014.https://pure.mpg.de/pubman/faces/ViewItemOverviewPage.jsp?itemId=item_2327525 (accessed 16 May2022).

12 Treiber JM, White NS, Steed TC, Bartsch H, Holland D, Farid N *et al.* Characterization and Correction of Geometric Distortions in 814 Diffusion Weighted Images. *PLOS ONE* 2016; **11**: e0152472.

13 Abraham A, Pedregosa F, Eickenberg M, Gervais P, Mueller A, Kossaifi J *et al.* Machine learning for neuroimaging with scikit-learn. *Front Neuroinformatics* 2014; **8**.https://www.frontiersin.org/article/10.3389/fninf.2014.00014 (accessed 19 Apr2022).

14 Garyfallidis E, Brett M, Amirbekian B, Rokem A, Van Der Walt S, Descoteaux M *et al.* Dipy, a library for the analysis of diffusion MRI data. *Front Neuroinformatics* 2014; **8**.https://www.frontiersin.org/article/10.3389/fninf.2014.00008 (accessed 19 Apr2022).

15 Avants B, Tustison NJ, Song G. Advanced Normalization Tools: V1.0. *Insight J* 2009. doi:10.54294/uvnhin.

16 Jenkinson M, Beckmann CF, Behrens TEJ, Woolrich MW, Smith SM. FSL. *NeuroImage* 2012; **62**: 782–790.

17 Yeh F-C. Population-based tract-to-region connectome of the human brain and its hierarchical topology. *Nat Commun* 2022; **13**: 4933.

18 Glasser MF, Smith SM, Marcus DS, Andersson JLR, Auerbach EJ, Behrens TEJ *et al.* The Human Connectome Project’s neuroimaging approach. *Nat Neurosci* 2016; **19**: 1175–1187.
